# An Integrated Network Pharmacology, Molecular Docking, Molecular Dynamics Simulation, and Experimental Validation Study to Investigate the Potential Mechanism of Isoliquiritigenin in the Treatment of Ischemic Stroke

**DOI:** 10.3390/cimb47080627

**Published:** 2025-08-06

**Authors:** Hang Yuan, Yuting Hou, Yuan Jiao, Xin Lu, Liang Liu

**Affiliations:** 1 Institute of Translational Medicine, Medical College, Yangzhou University, Yangzhou 225009, China; yzjsnyx@163.com (H.Y.); houytd@hotmail.com (Y.H.); 19552398538@163.com (Y.J.); luxin94lx@163.com (X.L.); 2 Key Laboratory of the Jiangsu Higher Education Institutions for Integrated Traditional Chinese and Western Medicine in Senile Diseases Control, Yangzhou University, Yangzhou 225009, China

**Keywords:** isoliquiritigenin, ischemic stroke, network pharmacology, molecular docking, molecular dynamics simulation, experimental validation

## Abstract

Isoliquiritigenin (ISL) is a type of chalcone that widely exists in medicinal plants of the Leguminosae family and exhibits a remarkable anti-ischemic stroke (IS) effect. However, the anti-IS mechanisms of ISL remain to be systematically elucidated. In this study, network pharmacology was used to predict potential targets related to the anti-IS effect of ISL. The binding ability of ISL to potential core targets was further analyzed by molecular docking and molecular dynamics (MD) simulations. By establishing an oxygen–glucose deprivation/reoxygenation (OGD/R)-induced HT22 cell model, the anti-IS mechanisms of ISL were investigated via RT-qPCR and Western Blot (WB). As a result, network pharmacology analysis revealed that APP, ESR1, MAO-A, PTGS2, and EGFR may be potential core targets of ISL for anti-IS treatment. Molecular docking and molecular dynamics simulation results revealed that ISL can stably bind to the five potential core targets and form stable complex systems with them. The results of the cell experiments revealed a significant anti-IS effect of ISL. Additionally, mRNA and protein expression levels of APP, MAO-A and PTGS2 or ESR1 in the ISL treatment group were significantly lower or higher than those in the OGD/R group In conclusion, ISL may improve IS by regulating the protein expression levels of APP, ESR1, MAO-A, and PTGS2.

## 1. Introduction

Stroke, which is one of the foremost causes of mortality worldwide, imposes a significant health burden [1]. Stroke can be divided into two primary types: hemorrhagic stroke and ischemic stroke (IS); moreover, IS accounts for more than 80% of all stroke cases [2]. IS is characterized by high incidence rates, substantial complications, elevated mortality, and frequent recurrence [3]. Modern medicine holds that the majority of ischemic strokes originate from thromboembolism. The brain is a high-oxygen-consuming organ. The deprivation of oxygen and glucose in local brain tissue caused by ischemia not only leads to the permanent loss of brain nerve function in a short period of time, but also causes a sharp decline in cellular energy production [4]. Current treatment strategies primarily include thrombolytic therapy, anticoagulation agents, neuroprotective agents, and surgical interventions [5]. However, due to the suddenness of the disease and the limitations of treatment conditions, as well as serious adverse reactions and many nonnegligible flaws, the clinical efficacy of these treatment methods is not satisfactory. Therefore, the search for new anti-IS drugs is extremely important.

Active compounds from medicinal plants have consistently been important resources for the development of innovative drugs [6,7]. In recent years, the research on medicinal plants in the treatment of IS injury has gradually deepened. Many studies have shown that the active components of medicinal plants can act on pathophysiological processes such as neuroinflammation, blood–brain barrier (BBB) injury, oxidative stress, and apoptosis; regulate their development; and effectively alleviate cerebral ischemic injury [8,9]. Many studies have focused on the significant neuroprotective effects of flavonoids [10]. Chalcones are a subclass of flavonoids; however, compared with other flavonoid subclasses, less attention has been given to chalcones [11]. Isoliquiritigenin (ISL) is a type of chalcones that widely exists in many medicinal plants of the Leguminosae family, including *Glycyrrhiza uralensis* Fisch., *Astragalus membranaceus* (Fisch.) Bunge, *Caragana jubata* (Pall.) Poir., and *C. changduensis* Y. X. Liou [12,13,14,15]. ISL has shown potential in treating multiple diseases. It can inhibit fatty acid synthesis and regulate the ERK signaling pathway, thereby suppressing the M2 polarization of macrophages and improving pulmonary fibrosis [16]. It ameliorates sarcomere contraction and inflammatory responses by inhibiting the IL-17RA/Act1/p38 signaling pathway in rats with myofascial trigger points [17]. And ISL can improve energy metabolism and antioxidant enzyme activity to relieve ischemic injury in rats [18]. However, the underlying molecular mechanisms of ISL in the treatment of IS remain to be fully investigated.

Network pharmacology is a new discipline that reveals the regulatory network of drugs in the body at the system level, and it aims to predict the mechanism of action of drugs by constructing a complex network of relationships among diseases, targets, and drugs [19]. This method is characterized by a high degree of integrity, systematization, and comprehensiveness; moreover, it is an effective method for revealing the pharmacological mechanisms of natural compounds. It employs big data and artificial intelligence to rapidly identify key targets and signaling pathways in complex biological systems. And it integrates large-scale data, network analysis, and algorithm optimization for studying drug mechanisms, with its research outcomes progressively translating into clinical applications and novel therapeutics [20]. Molecular docking and molecular dynamics (MD) simulations have emerged as characterization and virtual screening tools, due to the fact that they can visualize interaction details that cannot be identified by in vitro experiments and can quickly screen for bioactive compounds from large databases containing millions of molecules [21,22]. In this study, an integrated network pharmacology, molecular docking, MD simulation, and experimental validation study was performed to investigate the potential mechanism of isoliquiritigenin in the treatment of IS, thereby providing a theoretical basis for its further study and clinical application.

## 2. Materials and Methods

### 2.1. Materials and Reagents

Isoliquiritigenin (A0463) was purchased from Chengdu Must Biotechnology Co., Ltd. (Chengdu, China). The purities of the standards were above 98%. HT22 mouse hippocampal neurons and FBS were purchased from Pricella Biotechnology Co., Ltd. (Wuhan, China). DMEM (12100), DMSO (D8371) and phosphate-buffered saline (PBS) (P1020) were supplied by Beijing Solarbio Science & Technology Co., Ltd. (Beijing, China). Cell Counting Kit-8 (CCK-8) (KTA1020) was purchased from Abbkine Scientific Co., Ltd. (Wuhan, China). TRIzol reagent was purchased from Ambion (Austin, TX, USA). The PrimeScript™ RT reagent kit was purchased from TaKaRa (Otsu, Japan). The NovoStart^®^ SYBR qPCR SuperMix Plus kit (E096-01A) was purchased from Novoprotein (Shanghai, China). All of the primers were synthesized by Sangon Biotech Co., Ltd. (Shanghai, China). BCA protein assay kit was purchased from Beyotime Biotech Inc (Shanghai, China). APP (25524-1-AP), PTGS2 (12375-1-AP), and EGFR (30847-1-AP) antibodies were purchased from Proteintech Group, Inc. (Wuhan, China). The positive control drug edaravone (89-25-8), MAO-A antibody (YA2036) and ESR1 (YA3629) antibody were purchased from MedChemExpress LLC (Shanghai, China). HRP-conjugated goat anti-rabbit IgG (H + L) (AS014) was purchased from ABclonal Technology Co., Ltd. (Wuhan, China). PVDF membranes (cat. FFP24) and SDS-PAGE gels were purchased from Beyotime (Shanghai, China). The automated chemiluminescence imaging system (Tanon-4600) was manufactured by Tanon Science & Technology Co., Ltd. (Shanghai, China). The CFX96 Touch RealTime PCR Detection System was from Bio-Rad Laboratories, Inc. (Hercules, CA, USA). The microplate reader (Spark^®^) was from Tecan Group Ltd., (Männedorf, Switzerland).

### 2.2. Network Pharmacology Analysis

Potential targets of ISL were predicted using three in silico platforms: SwissTargetPrediction (http://www.swisstargetprediction.ch/, accessed on 12 April 2025), PharmMapper (http://www.lilab-ecust.cn/pharmmapper/, accessed on 12 April 2025), and the Similarity Ensemble Approach (SEA, http://sea.bkslab.org/, accessed on 12 April 2025), with species constraints rigorously set to *Homo sapiens*. To improve the reliability of the results, the targets were screened based on probability >0, fit score >3, and Max Tc >0.5. Disease targets were obtained from the GeneCards database (https://www.genecards.org/, accessed on 12 April 2025) and searched by using “Ischemic stroke” as the keyword [23]. The chemical constituent targets and disease targets were imported into Venny 2.1.0; additionally, the overlapping targets between ISL and IS were identified as potential therapeutic targets of ISL against IS, and the corresponding Venn diagram was drawn. The intersecting genes were subsequently uploaded to the STRING database (https://string-db.org/, accessed on 12 April 2025), with the species parameter set to “*Homo sapiens*”, in order to construct the protein–protein interaction (PPI) network [24]. The results were imported into Cytoscape 3.9.1 software for network construction and visual analysis [25]. The degree centrality, betweenness centrality, and closeness centrality of each target node were calculated using the cytoHubba plugin as evaluation metrics for the network analysis to identify the potential core anti-IS targets for ISL. Additionally, the intersecting genes were input into the DAVID database (https://david.ncifcrf.gov/, accessed on 12 April 2025) for Gene Ontology (GO) and Kyoto Encyclopedia of Genes and Genomes (KEGG) enrichment analyses [26]. A visual representation of the enrichment results was subsequently generated.

### 2.3. Molecular Docking

The selected protein crystal structures were sorted according to comprehensive analyses of degree centrality, betweenness centrality, and closeness centrality, and molecular docking with ISL was performed. The two-dimensional structure of ISL was downloaded from the TCMSP database (https://www.tcmsp-e.com, accessed on 12 April 2025). The three-dimensional structure of the core protein was downloaded in PDB format from the Research Collaboratory for Structural Bioinformatics Protein Data Bank (RCSB PDB) (https://www.rcsb.org/, accessed on 12 April 2025). After water molecules and ligands were removed from the protein via PyMOL 2.5.7 software, the 3D structure was used as a receptor for visualization of the molecular docking features. Docking grids were constructed as binding sites for the receptor, and the grid size was determined to be 40 × 40 × 40 A. Molecular docking of ligands and receptors was performed using AutoDock Vina 1.1.2 software.

### 2.4. MD Simulation

After molecular docking, the stability of the chosen docking poses was confirmed by MD by using Gromacs 2022 software. The charmm36-jul2021 force field with the TIP3P water model was used for the protein simulations, whereas the GAFF force field was applied for small molecules. The total charge of the system was neutralized by adding a suitable number of Na^+^ or Cl^−^ ions. The steepest-descent method was used for energy minimization. After energy minimization, equilibrium simulations were performed using both isothermal isovolumic (NVT) and isothermal isobaric (NPT) ensembles. MD simulations were subsequently conducted for 100 ns, which consisted of 5 million steps with a step length of 2 fs at 300 K and 1 atmosphere of pressure. Upon completion of the simulation, the gmx tools of Gromacs 2022 software were used to analyze the trajectory. Moreover, the Root-Mean-Square Deviation (RMSD), interaction fraction, and Gibbs free energy were calculated.

### 2.5. Cell Culture and Treatment

HT22 cells were cultured in DMEM supplemented with 10% FBS and 1% penicillin–streptomycin antibiotics (100 U/mL penicillin and 100 μg/mL streptomycin), and maintained at 37 °C in a 5% CO_2_ incubator. The following three experimental groups were established: the control group, the oxygen–glucose deprivation/reoxygenation (OGD/R) group, and the ISL group.

### 2.6. Cytotoxicity Evaluation of ISL

HT22 cells in the logarithmic growth phase were seeded in 96-well plates at a density of 1 × 10^4^ cells/well and cultured at 37 °C in a 5% CO_2_ incubator for 24 h. ISL was subsequently added to the cells for another 24 h or 72 h. Afterwards, DMEM culture solution containing 10% CCK-8 was added to the cells, and the absorbance of each cell at 450 nm was measured with a microplate reader after incubation for 1 h. Control group received an equal volume of culture medium containing 0.01% DMSO without ISL. The final concentrations of ISLwere 6.25, 12.5, 25, 50, 100, and 200 µM.

### 2.7. OGD/R Model Establishment

The in vitro neuroprotective effect of ISL was evaluated by establishing an OGD/R model. HT22 cells in the logarithmic growth phase were seeded in 96-well plates at a density of 1 × 10^4^ cells/well. After the cells were attached to the walls, they were washed twice with PBS, followed by incubation with ISL or edaravone (5 μM) for 24 h. Afterwards, sugar-free DMEM was added, and the plate was placed into an incubator containing 5% CO_2_, 1% O_2_, and 94% N_2_. After being deprived of glucose and oxygen for 4 h, the medium of the cells was replaced with sugar-containing complete medium, and the cells were cultured under normal oxygen conditions (95% O_2_ and 5% CO_2_) for 12 h. Following the addition of DMEM supplemented with 10% CCK-8 to the cells, the absorbance at 450 nm was measured. The control group was supplemented with an equal volume of culture medium without ISL and cultured at 37 °C in a 5% CO_2_ incubator throughout the experiment. In the OGD/R group, an equal volume of culture medium without ISL was added, and the cells were cultured under the same conditions as those in the ISL group. The final concentrations of ISL were 1.25, 2.5, 5, 10, and 20 μM. Edaravone served as a positive control, and its final concentration was 5 μM.

### 2.8. RT-qPCR Assay

Total RNA was extracted using the TRIzol reagent according to the manufacturer’s protocol from the following four groups of HT22 cells: control group, vehicle control group (0.01% DMSO), OGD/R group, and OGD/R+ISL group. We performed reverse transcription into cDNA using the PrimeScript™ RT reagent kit. Quantitative PCR (qPCR) analyses were performed using the NovoStart^®^ SYBR qPCR SuperMix Plus kit on the CFX96 Touch RealTime PCR Detection System (Bio-Rad, Hercules, CA, USA). The expression levels of the target genes were quantified using the 2^−ΔΔCT^ method, and the final results were normalized to those of GAPDH. The utilized primers for the qRT-PCR assays are listed in Table 1.

### 2.9. Western Blotting (WB)

Protein was extracted from three experimental groups of HT22 cells using RIPA lysis buffer containing protease and phosphatase inhibitors: control group, OGD/R group, and OGD/R+ISL group. Protein concentrations were quantified using a BCA protein assay kit, after which 20 μg of protein from each sample was electrophoresed on SDS-PAGE gels and subsequently transferred to PVDF membranes, which were blocked with 5% nonfat milk in TBST buffer for 1 h at room temperature and subsequently incubated with primary antibodies overnight at 4 °C. The primary antibodies used in this study included APP, ESR1, MAO-A, PTGS2, EGFR, β-tubulin, and GAPDH. The PVDF membranes were washed with TBST three times and incubated with HRP-conjugated secondary antibodies for 1 h at room temperature. The protein bands were then detected by enhanced chemiluminescence (ECL) using an automated chemiluminescence imaging system (Tanon-4600; Tanon Science & Technology Co., Shanghai, China). The intensity of the blots was quantified via ImageJ 1.54g software [27], and the obtained values were statistically analyzed.

### 2.10. Statistical Analysis

Statistical analyses were performed by using GraphPad Prism 9.0 software. All treatment allocations used computer-generated randomization. Biological replicates (*n* ≥ 3 independent cultures) with technical replicates averaged per experiment are specified in the figure legends. The Shapiro–Wilk test verified the normality assumption. Homogeneity of variances was confirmed by Levene’s test. Differences between two groups were assessed using the *t*-test, whereas comparisons among multiple groups were performed using one-way analysis of variance followed by Dunnett post hoc test. The data are presented as the means ± SDs. A value of *p* < 0.05 was considered statistically significant.

## 3. Results

### 3.1. Potential Targets of ISL in the Treatment of IS

**Figure 1:**
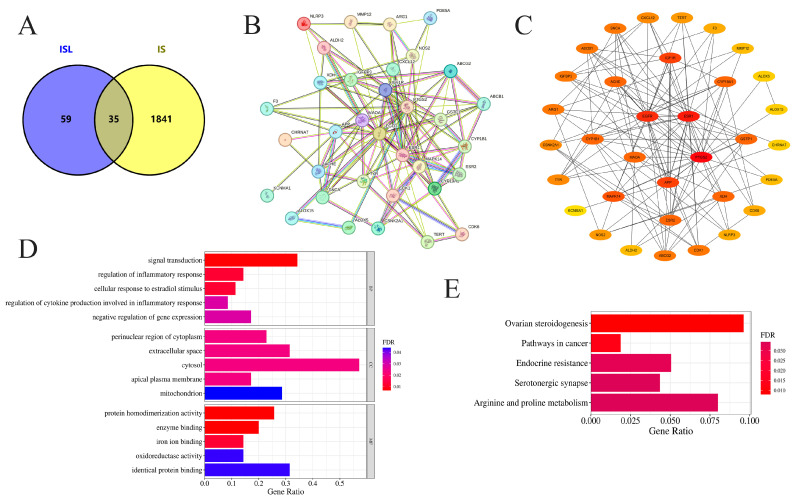
Network pharmacology analysis of the effects of ISL on IS. (**A**) Venn diagram visualization of target acquisition for ISL in the treatment of IS. (**B**) The PPI network of potential targets of ISL in the treatment of IS. (**C**) The key protein targets for ISL in the treatment of IS were ranked according to degree centrality, betweenness centrality, and closeness centrality. (**D**) GO-BP, GO-CC, and GO-MF enrichment analysis of potential targets. (**E**) KEGG enrichment and analysis of the top 5 key signaling pathways associated with potential targets. Bars show gene ratios and colors indicate FDR significance.

A total of 94 potential targets of ISL were identified through SwissTargetPrediction, PharmMapper, and SEA databases, while 1876 disease targets of IS were obtained from the GeneCards database (Table S1). The overlapping targets between ISL and IS were identified as potential targets. After removing duplicates, 35 intersecting genes were ultimately obtained, and the generated Venn diagram is shown in Figure 1A.

### 3.2. PPI Network Analysis and Screening of Key Targets

The 35 intersecting genes were imported into the STRING database, and the results were imported into Cytoscape 3.9.1 software for network construction and visual analysis, with the unconnected nodes in the network being hidden (Figure 1B). The PPI network comprised 34 nodes and 125 edges, thus reflecting the interactions between these potential ISL genes in the treatment of IS, which were screened via the cytoHubba plugin according to degree centrality, betweenness centrality, and closeness centrality (Table 2). Through the comprehensive considerations of these three parameters, the core targets might be PTGS2, ESR1, APP, EGFR, and MAO-A (Figure 1C).

### 3.3. GO and KEGG Enrichment Analyses

GO and KEGG functional enrichment analyses of the intersecting genes were conducted using the DAVID database, followed by visualization of the results. Ultimately, we obtained 139 biological process (BP), 27 cellular component (CC), and 63 molecular function (MF) terms based on the GO entry (Table S2). The top 5 terms in each category are shown in Figure 1D. In addition, KEGG enrichment analysis identified 39 pathways (Table S3). The top five enriched pathways suggest that ISL may affect IS via multiple signaling pathways (Figure 1E). KEGG enrichment analysis reveals potential signaling associations, though these pathways remain unvalidated functionally.

### 3.4. Molecular Docking Analysis

**Figure 2:**
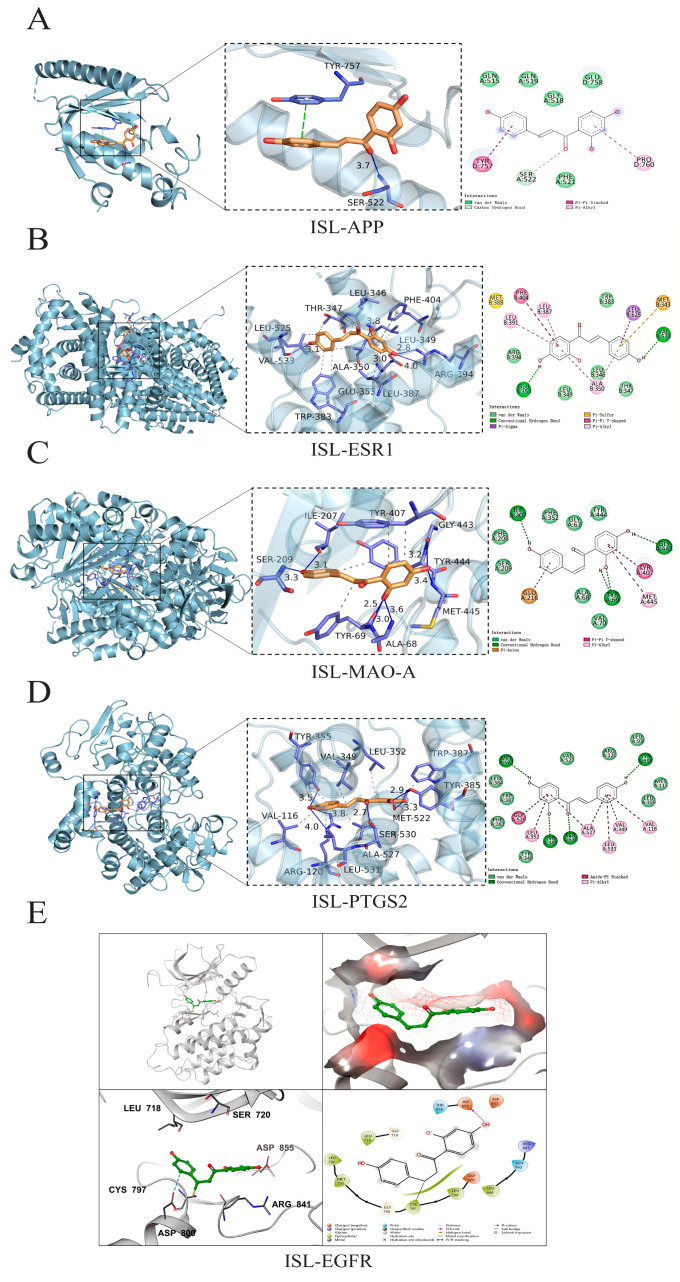
The molecular docking of ISL with the APP, ESR1, MAO-A, PTGS2, and EGFR proteins. The three-dimensional and 2D intermolecular contact between ISL and APP (**A**), ISL and ESR1 (**B**), ISL and MAO-A (**C**), ISL and PTGS2 (**D**), and ISL and EGFR (**E**).

The top five potential core targets, including APP (PDB: 3SV1), ESR1 (PDB: 7UJM), MAO-A (PDB: 2BXR), PTGS2 (PDB: 5IKR), and EGFR (PDB: 3IKA), were docked with ISL to clarify their possible binding modes and molecular interactions. The docking results were visualized, and the results are shown in Figure 2. ISL was able to effectively bind to the active pocket of the protein, indicating that the compound can undergo hydrogen bond interactions and hydrophobic interactions with the protein binding pocket. These interactions promote the binding of ISL to the active pocket of the protein to form a complex. When the binding affinity is <0 kcal·mol^−1^, the molecule and protein could be automatically bound; additionally, a lower binding affinity corresponds to a more stable binding conformation [28]. The molecular docking results revealed that the binding affinities of ISL for APP, ESR1, MAO-A, PTGS2, and EGFR were −5.80, −8.60, −8.80, −8.40, and −7.90 kcal·mol^−1^, respectively. For the targets with available co-crystal structures including ESR1, MAO-A, PTGS2, and EGFR, re-docking validation achieved RMSD values of 1.019 Å, 1.275 Å, 1.799 Å, and 1.677 Å, respectively (Table 3), indicating that ISL could stably bind to the key core targets.

### 3.5. MD Simulations Results

**Figure 3:**
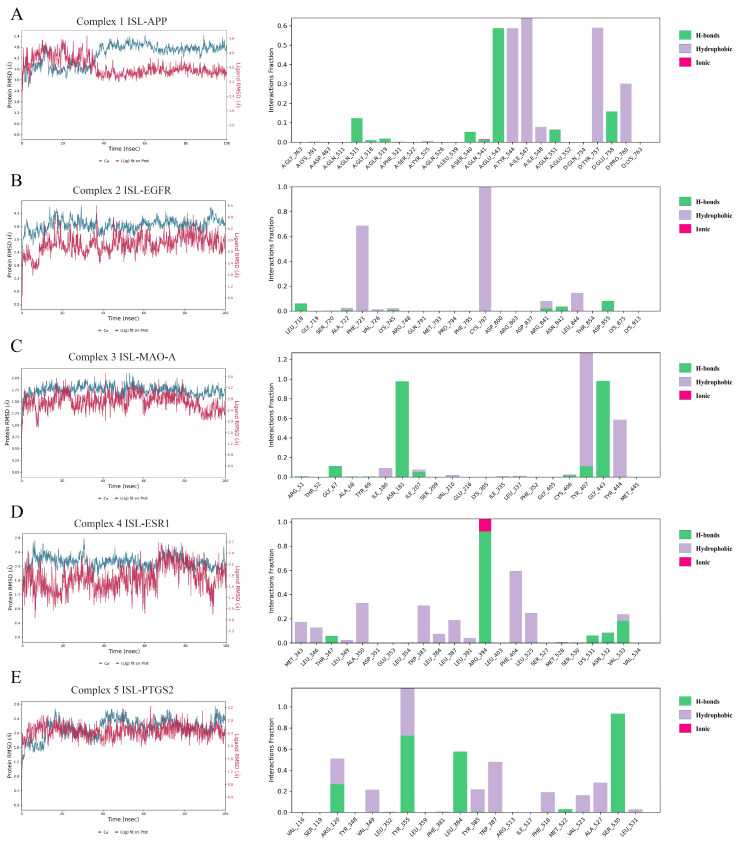
Molecular dynamics (MD) simulations of the ISL-APP (**A**), ISL-EGFR (**B**), ISL-MAO-A (**C**), ISL-ESR1 (**D**), and ISL-PTGS2 (**E**) complexes.

To further evaluate the influence of ISL on targets, MD simulations were conducted to determine the stability of the ISL-APP (Complex 1), ISL-EGFR (Complex 2), ISL-MAO-A (Complex 3), ISL-ESR1 (Complex 4), and ISL-PTGS2 (Complex 5) complexes obtained from molecular docking in 100 ns. The RMSD is a statistical method used to evaluate the stability of a simulated system. As shown in Figure 3, Complexes 1, 2, 3, 4, and 5 reached equilibrium after 40 ns, 20 ns, 20 ns, 20 ns, and 40 ns, respectively. Meanwhile, the RMSD of protein fluctuated between 3.0 Å and 5.5 Å, 3 Å and 4 Å, 1.5 Å and 2 Å, 2 Å and 2.5 Å, and 2 Å and 2.6 Å, respectively. These results indicated that ISL could generate a stable complex system with APP, EGFR, MAO-A, ESR1, and PTGS2, respectively, which was consistent with the molecular docking results. The MM-GBSA ΔG_bind_ was calculated and is listed in Table 4. The ΔG_bind_ was −41.3 ± 3.4, −40.9 ± 5.1, −46.9 ± 6.2, −31.7 ± 3.7, and −26.4 ± 5.0, respectively. As shown in Figure 3, for ISL-APP (Complex 1), Glu543, Tyr544, Ile547, and Tyr75 were important residues for binding. For ISL-EGFR (Complex 2), the covalent bond with Cys797 was vital; in addition, the hydrophobic interaction with Phe723 stabilized the complex as well. For ISL-MAO-A (Complex 3), the H-bond with Asn181 and Gly443, as well as the hydrophobic interaction with Tyr407 and Tyr444, contributed to ΔG_bind_. For ISL-ESR1 (Complex 4), Arg394 formed H-bonds or ionic interactions via the MD. The hydrophobic interactions with Phe404 and Trp383 further stabilized the complex. For ISL-PTGS2 (Complex 5), Tyr355 and Ser540 were most important. Arg120, Leu484, and Trp387 also made a contribution.

### 3.6. Effects of ISL on OGD/R-Induced HT22 Cells

**Figure 4:**
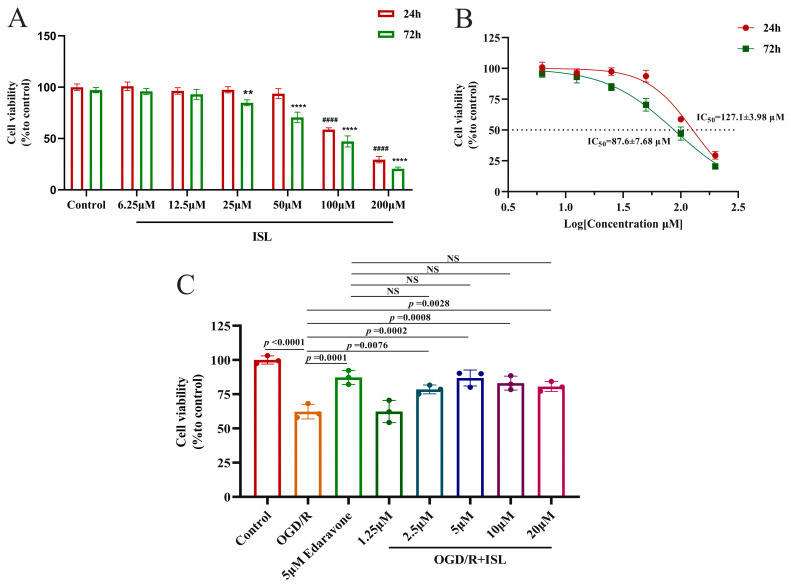
The neuroprotective effect of ISL on OGD/R-induced HT22 cells. Data are presented as the mean ± SD (*n* = 3). (**A**,**B**) The toxic effect of ISL (24 h/72 h) on HT22 cells under normal culture conditions. ^####^ *p* < 0.0001 vs. the control group at 24 h. ** *p* < 0.01 and **** *p* < 0.0001 vs. the control group at 72 h. (**C**) The effect of ISL on OGD/R-induced HT22 cells.

Within 24 h, ISL showed no obvious damage at the concentrations ranging from 6.25 to 50 µM, while 100 and 200 µM ISL exhibited toxicity (*p* < 0.0001). At 72 h, no obvious damage was observed at the concentrations of 6.25 and 12.5 µM, while ISL exhibited toxicity at the concentrations ranging from 25 to 200 µM (*p* < 0.01, *p* < 0.0001) (Figure 4A). The IC_50_ values at 24 h and 72 h were 127.1 ± 3.98 and 87.6 ± 7.68 µM, respectively (Figure 4B). Afterwards, the effect of ISL on OGD/R-induced HT22 cells was evaluated, and edaravone served as a positive control. As shown in Figure 4C, the cell viability of the OGD/R group was significantly lower than that of the control group (*p* < 0.0001), whereas ISL significantly increased the viability of HT22 cells induced by OGD/R at the concentrations ranging from 2.5 to 20 µM (*p* < 0.01, *p* < 0.001), indicating that ISL exerted an obviously protective effect on OGD/R-induced HT22 cells at the abovementioned concentration range. More importantly, 2.5–20 µM ISL exhibited effects comparable to those of 5 μM edaravone (*p* > 0.05). However, 1.25 µM ISL had no protective effects on OGD/R-induced HT22 cells, likely due to an insufficient concentration to reach the effective therapeutic threshold.

### 3.7. The Effect of ISL on the mRNA Expression of APP, ESR1, MAO-A, PTGS2, and EGFR

**Figure 5:**
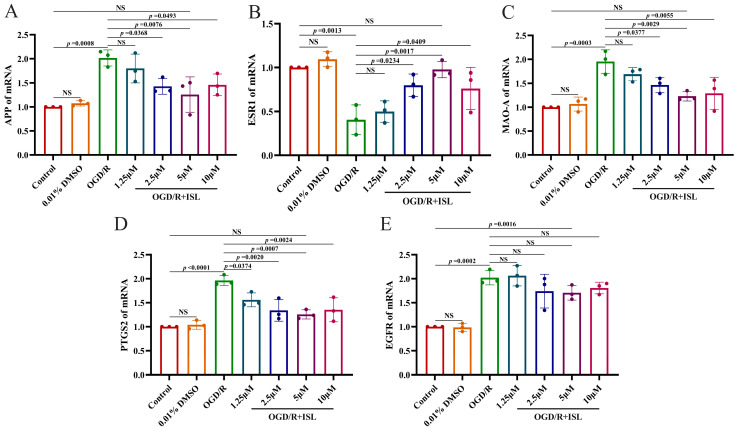
RT-qPCR analysis of the mRNA levels of APP (**A**), ESR1 (**B**), MAO-A (**C**), PTGS2 (**D**), and EGFR (**E**). Data are presented as the means ± SD (*n* = 3).

To further confirm the mechanisms of ISL against IS, we subsequently evaluated the mRNA levels of APP, ESR1, MAO-A, PTGS2, and EGFR after ISL treatment via RT-qPCR. As shown in Figure 5, ISL treatment significantly reduced the mRNA level of PTGS2 at the concentrations of 1.25–10 μM (*p* < 0.05, *p* < 0.01, and *p* < 0.001). It also decreased the mRNA levels of APP and MAO-A (*p* < 0.05, *p* < 0.01) and increased that of ESR1 (*p* < 0.05, *p* < 0.01) at the concentrations ranging from 2.5 to 10 μM, compared with those in the OGD/R group. Notably, when compared to the control group, the 5 μM ISL treatment group showed no significant differences in APP, ESR1, MAO-A, and PTGS2 expression levels (*p* > 0.05), suggesting a trend toward normalization. ISL treatment did not influence the mRNA level of EGFR at the tested concentrations. Additionally, there was no significant difference between the control group and the 0.01% DMSO group, which showed that the usage of 0.01% DMSO was nontoxic.

### 3.8. The Effect of ISL on the Protein Expression of APP, ESR1, MAO-A, PTGS2, and EGFR

**Figure 6:**
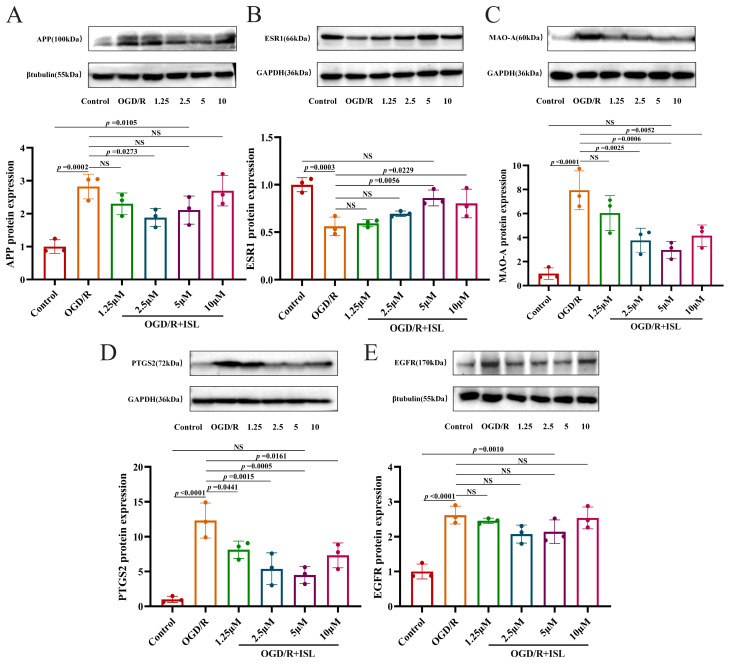
Effects of ISL on the protein expression levels of APP (**A**), ESR1 (**B**), MAO-A (**C**), PTGS2 (**D**), and EGFR (**E**) in HT22 cells. Data are presented as the means ± SD (*n* = 3).

As shown in Figure 6A–E, APP, MAO-A, PTGS2, and EGFR protein expression levels were significantly greater in the OGD/R model group than in the control group (*p* < 0.001 and *p* < 0.0001). And the expression level of ESR1 protein was markedly reduced relative to the control group (*p* < 0.001). Compared with the OGD/R group, ISL was able to significantly reduce the expression level of APP at the concentration of 2.5 μM (*p* < 0.05) and increase that of ESR1 protein at the concentrations of 5 and 10 μM, respectively (*p* < 0.05, *p* < 0.01). In addition, ISL significantly inhibited those of MAO-A and PTGS2 at the concentrations of 2.5–10 μM and 1.25–10 μM, respectively (*p* < 0.05, *p* < 0.01, and *p* < 0.001). Notably, ESR1, MAO-A, and PTGS2 protein expression levels of the 5 μM ISL treatment group were comparable to that of the control group (*p* > 0.05), suggesting a trend toward normalization.

## 4. Discussion

The pathogenesis of stroke mainly include cellular excitotoxicity, mitochondrial dysfunctions, neuroinflammation, BBB impairment, and cell death processes [29]. A previous study shows that ISL plays an important role in combating the pathological process of IS [30]. ISL has been proven to alleviate brain injury induced by ischemia in rats by inactivating the p38/MAPK pathway and promoting M2 polarization of microglia, thereby exerting an anti-IS effect. Meanwhile, ISL can improve tMCAO-induced brain injury by inhibiting autophagy and neuronal apoptosis and improving energy metabolism and its antioxidant properties [31,32]. However, there is still a lack of systematic research on its anti-IS characteristics at present. Therefore, we comprehensively investigated the mechanism of the anti-IS action of ISL by network pharmacology, molecular docking, molecular dynamics simulations, and experimental validation.

Based on the results of PPI network topology analysis, we can speculate that APP, ESR1, MAO-A, PTGS2, and EGFR are the main targets of ISL in the treatment of IS. Amyloid precursor protein (APP) is a universally expressed transmembrane protein, and previous studies have demonstrated that an increase in APP production can contribute to a cascade of cellular events that lead to neuronal dysfunction and death [33]. Beta amyloid (Aβ) is produced by the proteolytic cleavage of amino acids 695~770 of APP. A variety of cell types can produce Aβ from APP; moreover, in an acute ischemic model, ischemic brain injury was demonstrated to affect APP processing and lead to increased Aβ production [34]. Studies have demonstrated that the overexpression of APP increases the susceptibility of the brain to ischemic damage. This effect may involve transgene-derived Aβ neurotoxicity and Aβ-induced vascular dysfunction, thereby leading to more severe ischemia in areas at risk of infarction [35,36]. Prostaglandin-endoperoxidase synthase (PTGS2), which is also known as COX-2, is a major response gene involved in maintaining homeostasis in normal tissues and regulating inflammation [37]. The PTGS2 mRNA and protein are highly expressed in IS [38]. Studies have shown that PTGS2 reaction products are harmful to the ischemic brain. Moreover, the inhibition of PTGS2 by highly selective antagonists reduces ischemic damage in both focal and global cerebral ischemia models [39,40]. The genetic deletion of PTGS2 has been described as being an inhibitor of neuroinflammatory events to maintain the integrity of the BBB and mitigate IS-induced damage [41]. Monoamine oxidases (MAOs), including type A (MAO-A) and type B (MAO-B) MAOs in humans and mice, are a family of flavoenzymes associated with the outer mitochondrial membrane in neurons, astrocytes, and many other types of cells [42,43,44]. MAO produces large amounts of hydrogen peroxide, aldehydes, and ammonia during oxidative deamination, which can damage mitochondria and cause oxidative stress and neuronal death. Therefore, the inhibition of MAO activity exhibits important therapeutic value in the treatment of ischemic stroke-induced brain injury [45]. Estrogen receptor alpha (ESR1), a ligand-activated transcription factor, critically mediates transcriptional activation, hormone binding, and DNA binding, which regulates the expression of estrogen-responsive genes by interacting with estrogen [46]. Studies have shown that ESR1 can downregulate the expression of CYP1A1 and 20-HETE after stroke. And the posterior cortical 20-HETE levels were elevated in stroke patients and MCAO model mice. This indicates that ESR1 plays a crucial role in neuroprotection against hypoxic and ischemic damage [47,48]. The epidermal growth factor receptor (EGFR) serves as a critical therapeutic target for modulating pathological astrocyte overactivation. Following central nervous system (CNS) injury, EGFR expression undergoes rapid upregulation [49]. Evidence shows that p-PLCγ and EGFR expression is significantly upregulated following ischemia/reperfusion (I/R), suggesting a critical role for the EGFR/PLCγ signaling pathway in IS pathogenesis, thereby affecting neurons [50].

Molecular docking studies have shown that ISL has high affinities with the key targets identified by network pharmacology analyses. The results of MD simulations indicate that the bindings of ISL to the core targets are stable. Network pharmacology, molecular docking, and MD simulation rely on data and algorithms. However, due to the limitations of databases and software, it is impossible to fully capture the highly complex and dynamic nature of biological systems [51]. The combination of network pharmacology and in vitro experiments not only verified the accuracy of the prediction, but also provided deeper insights into the multi-target characteristics of natural drugs, thereby enhancing the scientific value and translational potential of the research [52].

Our following experimental results revealed that ISL can exert anti-IS effects by downregulating the mRNA and protein expression levels of APP, MAO-A, and PTGS2, and upregulating ESR1, which confirmed the results of the molecular docking and MD simulations. However, EGFR did not exhibit significant expression differences in the mRNA and protein levels, showing the necessity of verification experiments. These findings indicate that ISL exerts an anti-IS effect via a multitarget and multipathway pattern, which may involve the regulation of the inflammatory response, as well as cell and vascular renewal.

As demonstrated by the in vitro experiments, ISL has significant effects on the mRNA and protein levels of PTGS2 and MAO-A. Subsequent structural optimization of ISL can focus on improving their regulatory roles on PTGS2 and MAO-A. As shown in the results of molecular docking and MD simulations, the hydrogen bonds formed by OH are crucial for bindings. Therefore, the focus of structural optimization may be on the substituents of the benzene ring. In addition, the literature reports that the poor bioavailability and solubility in water of ISL have restricted its clinical application. It is a suitable strategy to make a prodrug to improve in vivo efficacy. Furthermore, proper nanodelivery technology can improve oral bioavailability or increase brain exposure [53]. This indicates that the development of brain-targeted ISL nanocarriers for the treatment of IS is very likely to improve the anti-IS effect of ISL.

## 5. Conclusions

In conclusion, ISL may exert an anti-IS effect by the regulation of APP, ESR1, MAO-A, and PTGS2, according to the combined results of network pharmacology, molecular docking, MD simulation, and experimental validation. All of the findings of the present study provide a theoretical basis for anti-IS drug development in the future.

## Figures and Tables

**Table 1:** Primers used in RT-qPCR.

Gene	Forward	Reverse
APP	TCCGAGAGGTGTGCTCTGAA	CCACATCCGCCGTAAAAGAATG
ESR1	AAGACGCTCTTGAACCAGCA	AGGCTTTGGTGTGAAGGGTC
MAO-A	GCCCAGTATCACAGGCCAC	CGGGCTTCCAGAACCAAGA
PTGS2	TGCACTATGGTTACAAAAGCTGG	TCAGGAAGCTCCTTATTTCCCTT
EGFR	CCGAAACTACGTGGTGACAGAT	TGCCATTACAAACTTTGCGAC
GAPDH	CACTCACGGCAAATTCAACGGCACA	GACTCCACGACATACTCAGCAC

**Table 2:** The corresponding parameters of the top ten targets ranked by degree value.

Gene Name	Degree	Betweenness Centrality	Closeness Centrality
PTGS2	24	320.2112364	28.5
ESR1	22	218.4691729	27.5
EGFR	19	119.9617377	26
APP	11	114.8404762	22
IGF1R	11	18.46190476	21.83333333
MAPK14	9	19.00417711	20.66666667
MAOA	8	26.03433584	20.5
XDH	8	33.59444444	20.33333333
ACHE	8	27.98145363	20.16666667
GSTP1	8	24.95873016	20.16666667

**Table 3:** Binding affinity and RMSD of ISL docked to potential core targets.

Target Protein	Binding Affinity (kcal·mol^−1^)	RMSD (Å)
APP	−5.80	/
ESR1	−8.60	1.019
MAO-A	−8.80	1.275
PTGS2	−8.40	1.799
EGFR	−7.90	1.677

“/” indicates no RMSD value available due to the absence of a co-crystal structure.

**Table 4:** The computed MM-GBSA ΔG_bind_ values (*n* = 3).

Protein	ΔG_bind_ (MM-GBSA)
APP	−41.3 ± 3.4
EGFR	−40.9 ± 5.1
MAO-A	−46.9 ± 6.2
ESR1	−31.7 ± 3.7
PTGS2	−26.4 ± 5.0

## Data Availability

The original contributions presented in this study are included in the article/Supplementary Materials. Further inquiries can be directed at the corresponding authors.

## Supplementary Materials

The following supporting information can be downloaded at https://www.mdpi.com/article/10.3390/cimb47080627/s1.

## References

[1] Zhang R., Lin Y.Q., Wang W.S., Wang X.Q. (2017). Excessive nNOS/NO/AMPK signaling activation mediated by the blockage of the CBS/H2S system contributes to oxygen-glucose deprivation-induced endoplasmic reticulum stress in PC12 cells. Int. J. Mol. Med..

[2] Paul S., Candelario-Jalil E. (2021). Emerging neuroprotective strategies for the treatment of ischemic stroke: An overview of clinical and preclinical studies. Exp. Neurol..

[3] Jin X., Wang R.-H., Wang H., Long C.-L., Wang H. (2015). Brain protection against ischemic stroke using choline as a new molecular bypass treatment. Acta Pharmacol. Sin..

[4] Soria F.N., Pérez-Samartín A., Martin A., Gona K.B., Llop J., Szczupak B., Chara J.C., Matute C., Domercq M. (2014). Extrasynaptic glutamate release through cystine/glutamate antiporter contributes to ischemic damage. J. Clin. Investig..

[5] Bindal P., Kumar V., Kapil L., Singh C., Singh A. (2024). Therapeutic management of ischemic stroke. N-S Arch. Pharmacol..

[6] Samkaria S., Kumari P. (2025). Wild Edible Flowers of Indian Himalayan Region, Their Traditional Uses and Potential Health Benefits: A Way Forward for Food and Nutritional Security. Plant Food Hum. Nutr..

[7] Wang H., Lu J., Chen X., Zhang K., Zhao X., Zhang Y. (2025). Antidiabetic and antioxidant molecules of *Ficus tikoua* and their combined effect: Bioassay-guided isolation, in vitro and in silico analysis. Plant Food Hum. Nutr..

[8] Hong Z., Cao J., Liu D., Liu M., Chen M., Zeng F., Qin Z., Wang J., Tao T. (2023). Celastrol targeting Nedd4 reduces Nrf2-mediated oxidative stress in astrocytes after ischemic stroke. J. Pharm. Anal..

[9] Younis N.S., Ghanim A.M.H. (2022). The Protective Role of Celastrol in Renal Ischemia-Reperfusion Injury by Activating Nrf2/HO-1, PI3K/AKT Signaling Pathways, Modulating NF-κb Signaling Pathways, and Inhibiting ERK Phosphorylation. Cell Biochem. Biophys..

[10] Subedi L., Gaire B.P. (2021). Phytochemicals as regulators of microglia/macrophages activation in cerebral ischemia. Pharmacol. Res..

[11] Zhou J., Sun F., Zhang W., Feng Z., Yang Y., Mei Z. (2024). Novel insight into the therapeutical potential of flavonoids from traditional Chinese medicine against cerebral ischemia/reperfusion injury. Front. Pharmacol..

[12] Lan X., Wang Q., Liu Y., You Q., Wei W., Zhu C., Hai D., Cai Z., Yu J., Zhang J. (2024). Isoliquiritigenin alleviates cerebral ischemia-reperfusion injury by reducing oxidative stress and ameliorating mitochondrial dysfunction via activating the Nrf2 pathway. Redox Biol..

[13] Cui L., Ma Z., Li W., Ma H., Guo S., Wang D., Niu Y. (2023). Inhibitory activity of flavonoids fraction from *Astragalus membranaceus* Fisch. ex Bunge stems and leaves on *Bacillus cereus* and its separation and purification. Front. Pharmacol..

[14] Zhao D., Chen X., Wang R., Pang H., Wang J., Liu L. (2023). Determining the chemical profile of *Caragana jubata* (Pall.) Poir. by UPLC–QTOF–MS analysis and evaluating its anti-ischemic stroke effects. J. Ethnopharmacol..

[15] Fang S.M. (2009). Chemical Constituents of Traditional Tibetan Medicine *Caragana changduensis*. Master’s Thesis.

[16] Mei X., Zhang Q., Shao W., Zheng J., Zhao P., Li J. (2025). Targeted Inhibition of Aldose Reductase by Isoliquiritigenin Suppresses Fatty Acid Synthesis to Inhibit Macrophage M2 Polarization and Alleviate Pulmonary Fibrosis. J. Agric. Food Chem..

[17] Li X., Qi T., Zhou L., Lin P., Chen Q., Li X., He R., Yang S., Liu Y., Qi F. (2025). Isoliquiritigenin alleviates abnormal sarcomere contraction and inflammation in myofascial trigger points via the IL-17RA/Act1/p38 pathway in rats. Phytomedicine.

[18] Zhan C., Yang J. (2006). Protective effects of isoliquiritigenin in transient middle cerebral artery occlusion-induced focal cerebral ischemia in rats. Pharmacol. Res..

[19] Liu X.L., Zhu P.Y., Ma J.R., Wang X.Y., Wei Q., Gao Y.M., Zhang D.Y., Yuan W.J. (2024). Antioxidant activity and mechanism of *Centranthera grandiflora* Benth roots based on network pharmacology and in vitro experiments. Nat. Prod. Res. Dev..

[20] Tao L., Ke Z.P., Wang T.J., Wang Z.Z., Cao L., Xiao W. (2025). Frontier technologies and development trends of network pharmacology: A patent bibliometric analysis. China J. Chin. Mater. Med..

[21] Surovi S., Manobjyoti B. (2019). Molecular Docking: Challenges, Advances and its Use in Drug Discovery Perspective. Curr. Drug Targets.

[22] Yu Y., Xu S., He R., Liang G. (2023). Application of Molecular Simulation Methods in Food Science: Status and Prospects. J. Agric. Food Chem..

[23] Stelzer G., Rosen N., Plaschkes I., Zimmerman S., Twik M., Fishilevich S., Stein T.I., Nudel R., Lieder I., Mazor Y. (2016). The GeneCards suite: From gene data mining to disease genome sequence analyses. Curr. Protoc. Bioinform..

[24] Szklarczyk D., Gable A.-L., Nastou K.-C., Lyon D., Kirsch R., Pyysalo S., Doncheva N.-T., Legeay M., Fang T., Bork P. (2021). The STRING database in 2021: Customizable protein-protein networks, and functional characterization of user-uploaded gene/measurement sets. Nucleic Acids Res..

[25] Otasek D., Morris J.-H., Bouças J., Pico A.-R., Demchak B. (2019). Cytoscape Automation: Empowering workflow-based network analysis. Genome Biol..

[26] Dennis G., Sherman B.-T., Hosack D.-A., Yang J., Gao W., Lane H.-C., Lempicki R.A. (2003). DAVID: Database for annotation, visualization, and integrated discovery. Genome Biol..

[27] Schneider C.A., Rasband W.S., Eliceiri K.W. (2012). NIH Image to ImageJ: 25 years of image analysis. Nat. Methods.

[28] Ouyang G., Zhu Y., Ouyang Z. (2025). Investigation of *Scutellaria Barbata*’s immunological mechanism against thyroid cancer using network pharmacology and experimental validation. Sci. Rep..

[29] Qin C., Yang S., Chu Y.-H., Zhang H., Pang X.-W., Chen L., Zhou L.-Q., Chen M., Tian D.-S., Wang W. (2022). Signaling pathways involved in ischemic stroke: Molecular mechanisms and therapeutic interventions. Signal Transduct. Target. Ther..

[30] He D., Wang P., Liao F., Yu L., Gan B. (2022). Cell membrane-coated biomimetic magnetic nanoparticles for the bio-specific extraction of components from Gualou Guizhi decoction exhibiting activities against oxygen-glucose deprivation/reperfusion injury. J. Pharm. Biomed. Anal..

[31] Wang R., Zhang W. (2023). Isoliquiritigenin regulates microglial M1/M2 polarisation by mediating the P38/MAPK pathway in cerebral stroke. J. Pharm. Pharmacol..

[32] Song W., Bai L., Yang Y., Wang Y., Xu P., Zhao Y., Zhou X., Li X., Xue M. (2022). Long-Circulation and Brain Targeted Isoliquiritigenin Micelle Nanoparticles: Formation, Characterization, Tissue Distribution, Pharmacokinetics and Effects for Ischemic Stroke. Int. J. Nanomed..

[33] Shi J., Yang S.-H., Stubley L., Day A.-L., Simpkins J.-W. (2000). Hypoperfusion induces overexpression of β-amyloid precursor protein mRNA in a focal ischemic rodent model. Brain Res..

[34] Nihashi T., Inao S., Kajita Y., Kawai T., Sugimoto T., Niwa M., Kabeya R., Hata N., Hayashi S., Yoshida J. (2001). Expression and Distribution of Beta Amyloid Precursor Protein and Beta Amyloid Peptide in Reactive Astrocytes After Transient Middle Cerebral Artery Occlusion. Acta Neurochir..

[35] Huang K.-L., Lin K.-J., Ho M.-Y., Chang Y.-J., Chang C.-H., Wey S.-P., Hsieh C.-J., Yen T.-C., Hsiao I.-T., Lee T.-H. (2012). Amyloid deposition after cerebral hypoperfusion: Evidenced on [^18^F]AV-45 positron emission tomography. J. Neurol. Sci..

[36] Zhang F., Eckman C., Younkin S., Hsiao K.-K., Iadecola C. (1997). Increased susceptibility to ischemic brain damage in transgenic mice overexpressing the amyloid precursor protein. J. Neurosci..

[37] Ma S.-L., Tang N.-L.-S., Zhang Y.-P., Ji L.-d., Tam C.-W.-C., Lui V.-W.-C., Chiu H.-F.-K., Lam L.-C.-W. (2008). Association of prostaglandin-endoperoxide synthase 2 (PTGS2) polymorphisms and Alzheimer’s disease in Chinese. Neurobiol. Aging.

[38] Cui Q., Zhang Y.-L., Ma Y.-H., Yu H.-Y., Zhao X.-Z., Zhang L.-H., Ge S.-Q., Zhang G.-W., Qin X.-D. (2020). A network pharmacology approach to investigate the mechanism of Shuxuening injection in the treatment of ischemic stroke. J. Ethnopharmacol..

[39] Nakayama M., Uchimura K., Zhu R.-L., Nagayama T., Rose M.-E., Stetler R.-A., Isakson P.-C., Chen J., Graham S.-H. (1998). Cyclooxygenase-2 inhibition prevents delayed death of CA1 hippocampal neurons following global ischemia. Proc. Natl. Acad. Sci. USA.

[40] Nogawa S., Zhang F., Ross M.-E., Iadecola C. (1997). Cyclooxygenase-2 gene expression in neurons contributes to ischemic brain damage. J. Neurosci..

[41] Yang C., Yang Y., DeMars K.-M., Rosenberg G.-A., Candelario-Jalil E. (2020). Genetic Deletion or Pharmacological Inhibition of Cyclooxygenase-2 Reduces Blood-Brain Barrier Damage in Experimental Ischemic Stroke. Front. Neurol..

[42] Shih J.-C., Chen K. (2004). Regulation of MAO-A and MAO-B Gene Expression. Curr. Med. Chem..

[43] Wang C.-C., Borchert A., Ugun-Klusek A., Tang L.-Y., Lui W.-T., Chu C.-Y., Billett E., Kuhn H., Ufer C. (2011). Monoamine Oxidase A Expression Is Vital for Embryonic Brain Development by Modulating Developmental Apoptosis. J. Biol. Chem..

[44] Tong J., Meyer J.-H., Furukawa Y., Boileau I., Chang L.-J., Wilson A.-A., Houle S. (2013). Distribution of Monoamine Oxidase Proteins in Human Brain: Implications for Brain Imaging Studies. J. Cereb. Blood Flow Metab..

[45] Deshwal S., Di S.-M., Di L.-F., Kaludercic N. (2017). Emerging role of monoamine oxidase as a therapeutic target for cardiovascular disease. Curr. Opin. Pharmacol..

[46] He P., Wang Z., Yang J., Pan P., Shi T., Xu S., Lan J., Hao Z., Yang A., Chen L. (2025). Mechanism of *Ligusticum wallichii*—Borneol in the Treatment of Cerebral Ischemic Stroke in Rats Based On Network Pharmacology, Molecular Docking, and Experimental Verification. Chem. Biodivers..

[47] Zhang Y., Jiao X., Qi X., Wang G., Ma Y. (2025). Edaravone ameliorates inflammation in ischemic stroke mouse by regulating the CYP1A1 pathway through gut microbiota. Exp. Neurol..

[48] Crago E.A., Thampatty B.P., Sherwood P.R., Kuo C.-W.J., Bender C., Balzer J., Horowitz M., Poloyac S.M. (2011). Cerebrospinal Fluid 20-HETE Is Associated with Delayed Cerebral Ischemia and Poor Outcomes After Aneurysmal Subarachnoid Hemorrhage. Stroke.

[49] Chen J., He W., Hu X., Shen Y., Cao J., Wei Z., Luan Y., He L., Jiang F., Tao Y. (2017). A role for ErbB signaling in the induction of reactive astrogliosis. Cell Discov..

[50] Ren Z.-L., Zhang C.-X., Zheng Y.-X., Chen C.-A., Dan C., Lan X., Yan X., Liu Y., He Y.-H., Cheng J.-L. (2025). Refined qingkailing attenuates reactive astrocytes and glial scar formation after ischemia stroke via the EGFR/PLCγ pathway. Phytomedicine.

[51] Luo T.-T., Lu Y., Yan S.-K., Xiao X., Rong X.-L., Guo J. (2020). Network Pharmacology in Research of Chinese Medicine Formula: Methodology, Application and Prospective. Chin. J. Integr. Med..

[52] Sun R., Liu C., Liu J., Yin S., Song R., Ma J., Cao G., Lu Y., Zhang G., Wu Z. (2023). Integrated network pharmacology and experimental validation to explore the mechanisms underlying naringenin treatment of chronic wounds. Sci. Rep..

[53] Zhang K., Wang Q., Yang Q., Wei Q., Man N., Adu-Frimpong M., Toreniyazov E., Ji H., Yu J., Xu X. (2019). Enhancement of Oral Bioavailability and Anti-hyperuricemic Activity of Isoliquiritigenin via Self-Microemulsifying Drug Delivery System. AAPS PharmSciTech.

