# Supplementary material for: An Integrated Network Pharmacology, Molecular Docking, Molecular Dynamics Simulation, and Experimental Validation Study to Investigate the Potential Mechanism of Isoliquiritigenin in the Treatment of Ischemic Stroke
Source: Curr Issues Mol Biol. 2025 Aug 6;47(8):627. doi: 10.3390/cimb47080627 (PMC12384097; doi:10.3390/cimb47080627)
Supplement: Supplementary file 1 [file cimb-47-00627-s001.zip › cimb-3716195-supplementary.pdf]

An integrated network pharmacology, molecular docking, molecular dynamics simulation, and experimental validation study to investigate the potential mechanism of isoliquiritigenin in the treatment of ischemic stroke

Hang Yuan [1,2], Yuting Hou[1,2], Yuan Jiao[1,2], Xin Lu[1,2] and Liang Liu[1,2,*]

1 Institute of Translational Medicine, Medical College, Yangzhou University, Yangzhou 225009, China

2 Key Laboratory of the Jiangsu Higher Education Institutions for Integrated Traditional Chinese and Western Medicine in Senile Diseases Control (Yangzhou University), Yangzhou, 225009, PR China

* Correspondence: enjoyyz@163.com (L.L.).

Table S1. Target Prediction Datasets.

IS targets	ISL targets	Overlapping targets
HDAC1	AKR1B1	CHRNA7
F2	CHRNA7	EGFR
ITGA4	EGFR	TERT
PLG	TERT	ABCG2
NMDAR2B	ABCG2	APP
PROC	APP	CYP19A1
BDKRB2	CYP19A1	MAOA
HCAR2	MAOA	F3
CPB2	F3	NOS2
TACR3	NOS2	SNCA
VWF	SNCA	ALDH2
F11	ALDH2	ALOX5
TARDBP	KCNA3	PTGS2
PDE1	BACE1	ABCB1
NMDAR	ALOX5	ESR1
OPR	PTGS2	ESR2
PARP	MAOB	ACHE
F5	PDPK1	PDE4D
STRK1	ABCB1	KCNMA1
NOS3	ESR1	IGF1R
ALOX5AP	ESR2	XDH
GP1BA	ACHE	CDK6
ACE	TLR9	CSNK2A1
THPH2	HSD17B2	CYP1B1
THPH1	HSD17B1	TYR
FLAP	PDE4D	IGFBP3
BSS	TUBB1	ALOX15
DCP1	KCNMA1	CDK1
RPRGL1	PTPRS	MMP12
RPRGL2	AMY1A	ARG1
BDPLT1	GRK6	MAPK14
ACE1	CTSL	GSTP1

VWDP	IGF1R	PDE5A
MVCD3	PTPN1	NLRP3
BDPLT3	NOX4	CXCL12
ICH	XDH	
MTHFR	FLT3	
NOTCH3	CCNB3	
COL4A1	CDK6	
MT-TL1	ABCC1	
GLA	CSNK2A1	
ADA2	CFTR	
HTRA1	CYP1B1	
BDNF-AS	TNKS2	
ITGB3	TNKS	
SERPINC1	ALOX12	
APOE	STS	
COL4A2	DHODH	
APP	FNTA	
CST3	TYR	
JAK2	AHR	
F7	IGFBP3	
PRKCH	PIM1	
FBN1	CBR1	
ENG	AKR1C3	
ABCC6	KDM4E	
CRP	ALOX15	
F13A1	CDK1	
MYH11	GLO1	
TREX1	MMP12	
FGB	CD38	
PDE4D	TOP1	
POLG	ARG1	
IL6	GSTA1	
APOB	SLC22A12	
RNF213	CDK2	
SERPINE1	MGAM	
TTR	ESRRB	
TNF	CCNB1	
FLNA	CCNB2	

HBB	FNTB
ITGA2B	MAPK14
LDLR	BCHE
KCNQ1	CDK5R1
ACVRL1	GSTP1
MT-TK	HCK
MT-ND1	CES1
F10	PDE5A
CDKN2B-AS1	CA2
GUCY1A1	AKR1B10
MALAT1	CA12
MIAT	CA14
CBS	CA1
MT-CYB	CA3
PLAT	CA4
MT-TS1	CA5A
KRIT1	CA5B
SLC2A10	CA6
SH2B3	CA7
IL10	CA9
MT-ND5	CYP1A2
MMP9	MYOC
PIK3CA	NLRP3
PDE3A	CXCL12
CFH	
FGA	
NPPA	
SMARCAL1	
CPS1	
PMM2	
HABP2	
PRKAG2	
MT-TI	
BDNF	
MEG3	
ALPL	
NPPB	
SELP	

ACTA2
MT-TL2
WFS1
IL1B
OTC
SOST
LOC132090228
HLA-DQB1
EPOR
POLGARF
STIM1
MT-CO1
PNP
PCNT
MT-TP
APOA1
VHL
MMUT
IGF1
MT-CO3
FGFR1
CD36
PON1
TP53
ASS1
SLC19A2
PCCA
MT-ND4
XYLT1
H19
IVD
MT-ND6
AGTR1
GYS1
ACAD9
ACSL4
MT-CO2
TRAPPC11

ELMO2
PCCB
INS
APOH
AGT
ERF
ZSWIM6
RBM8A
GFAP
GAS5
SPARC
PRF1
JAM3
PRKCG
EOGT
TMEM127
XYLT2
USP18
CFHR1
CFHR3
SCN5A
LPL
SNX14
RAB27A
SAMD9
ATRIP
MAX
SMAD4
MAPT
MT-ATP6
PPARG
TLR4
NDUFS1
HIF1A
HOTAIR
XRCC4
PIK3R5
F3

GALK1
ESR1
SELE
ICAM1
LMNA
CHD1
EBF3
PTGS2
ADIPOQ
SMAD2
THBD
CYP2C19
TNNI3
ADAMTS13
PTEN
NLRP3
CXCL8
EDN1
KRAS
LPA
CCL2
MIR155
VEGFA
CTLA4
ITGA2
ATRIP-TREX1
IL1A
KCNJ5
ACTB
AKT1
TGFBR1
TGFB1
LOX
SOD2-OT1
GRN
TGFBR2
RPL36A-HNRNPH2
SOD1

IL4
MIR125A
ELN
CERNA3
SLC2A1
MMP3
HLA-B
TNNT2
ALB
BMP7
MYBPC3
TUG1
ENO2
MIR146A
MIR140
MTR
CXCL12
IL18
SMAD3
MIR133B
BRAF
TNFRSF1A
FAS
IL1RN
SLC6A4
MIR21
CTNNB1
CASP3
REN
CSF1R
PDGFRB
HDAC9
COL3A1
KAT6B
MYLK
TREM2
MMP2
MIR126

CACNA1A
PLA2G7
MIR223
CDKN2A
XRCC1
MAPK1
VCAM1
MAPK3
CXCR4
MIR320A
MTRR
CALR
LTA
F12
IGF1R
NOTCH1
MTOR
TWIST1
JAG1
CCR5
HMGB1
ABCC9
ABCB1
NDE1
CCM2
CYP2C9
HMGCR
MYH7
ARG1
ADRB2
BCL2
SPP1
TTN
CDKN2B
TH
MIR143
MIR499A
S100B

FTO
VDR
TGFB3
MGP
PCSK9
MIR145
EPO
PRKG1
LAMP2
DNMT3A
PSEN1
RAF1
GBA1
KNG1
IL17A
UBE3A
MIR146B
PECAM1
FGF2
LEP
NOS2
HRAS
EGF
TIMP1
PITX2
MIR216A
B2M
TGFB2
MIR150
KDR
EIF2AK2
MAPK8IP1
NF1
GRIN2B
NKX2-5
PIK3C2A
ATP7B
CD40LG

MYH6
PVT1
CAV1
APOC3
SNHG12
MIR27B
PSEN2
ALDH2
CACNA1C
GP6
VKORC1
CETP
PTH
THPO
FGG
ENPP1
GATA6
RET
TMX2-CTNND1
MAP2K1
MIR144
ABCC8
SERPINA3
MIR17
NRAS
CREB1
UCP2
MIR451A
COMT
FGFR2
ABCA1
LINC01672
MIR149
ARSA
GRIP1
COG2
PARP1
MPO

MIR210
EPHX2
FADD
MMP1
MIR34C
COL5A1
MIR221
CD14
SLC1A3
MEFV
RYR2
HSPB1
DIAPH1
SLC1A2
SERPINA1
NOS1
MIR34A
HSPA4
PRTN3
GJA1
CBL
FGF23
LIPC
EP300
MIR29A
CDC42
HMOX1
GATA3
HTR1A
TNFRSF11B
F8
AKT2
PF4
PON2
SDHB
PHACTR1
PTGIS
PRNP

ITGB1
PRKN
EIF2AK3
FGFR3
MIR125B1
SERPINI1
CSF3
ZFHX3
CYCS
ROBO1
GATA4
CDK6
CYP11B2
PDCD10
APOA5
ALOX5
STAT3
TLR2
BRD4
FGF8
MIR211
XIST
EZH2
ACHE
PRKAR1A
CSF2
HGF
ACTC1
TET2
NAGA
SCN1B
RNF213-AS1
CRKL
FGF17
MIR532
SMAD5-AS1
HP
NPY

FLT1
CDC5L
PTGS1
ARHGEF6
MIR142
IL2
ANXA5
TSPO
AHSG
NGF
CD40
VPS11
MAPK8IP2
IL13
LRP1
SRC
SKI
PLA2G6
YAP1
GDF2
SDHA
MIR141
PPBP
KCNJ2
VCP
KCNMA1
SMARCA4
GCH1
MIR200B
IFNG
COL1A1
FOS
SLC9A1
NR3C2
TEK
BRCA2
DNAH8
OLR1

CCND2
SOX4
THBS1
CHAT
MAOA
SON
P2RY12
SLC17A5
MIR98
NTRK2
DMD
XDH
CCR2
TBXAS1
MIR199A1
SCN3B
PGR-AS1
CALM1
MPL
SOX2
SOS1
NEAT1
HSPA8
HFE
BRCC3
RETN
MIR503
GJA5
MIR574
MIR122
YY1
PSAP
ZEB2
HSPA1A
CYP4F2
SNHG1
AGER
PDGFB

GNAS
MYCN
COL2A1
AKT3
ACTG1
MIR124-1
CDK5
NFE2L2
EIF4E
CYP17A1
SETD2
GDNF
SDHC
MIR99A
ADD1
SIRT1
MIR9-1
MIR30A
FASN
MIR342
PWAR1
NFKB1
CTSA
ERBB4
SQSTM1
GNAQ
SLIT2
MIR193A
ACE2
MIR495
FOLR1
ASTN2
MIR130A
ERCC1
ARID1A
GAA
EDNRA
MIR18A

GPT
PTPN11
PARK7
ANK2
HOTTIP
MIR106B
GGCX
SOCS1
SYP
SCN4B
PRKDC
MIR19A
ADM
MIR134
USP7
CP
IFIH1
MIR206
AVP
PROZ
DLG4
HCN4
DNAJC6
MIR130B
ITM2B
ATP13A2
HSPD1
HK1
MIR215
GRIN2A
UCHL1
PANK2
FABP2
PPARA
AIFM1
PLOD1
MIR15A
NPC1

G6PD
LINC02605
SCNN1A
LMNB1
ADRB1
MIR185
MIR16-1
HBA1
COL1A2
MAPK8
ENTPD1
TRAP1
MMP7
PIGB
TNFSF10
GAL
CD163
DENND5A
MIR222
DBH
MIR877
JUN
BCL2L1
SIX2
UFM1
NECAP1
UFC1
DES
ITGAM
ATP1A3
CYP27A1
NPC2
VEGFC
DSP
SNHG16
AGTR2
IRF1
DPAGT1

ESR2
EPAS1
MIR485
SELPLG
MIR10B
ERCC4
GRIK2
FN1
WDR26
MIR501
NTNG1
MIR29B1
MIRLET7B
SOD2
BECN1
ECE1
ABCD1
CACNA2D1
CRH
LTC4S
AOC3
MIR411
MMACHC
CACNA1D
HTRA2
TF
IL1R1
MIR494
CYP3A4
CD63
UPK3A
NR3C1
MIR885
LCN2
ANGPT1
MDM2
CHI3L1
SNHG14

MIF
GAPDH
MIR152
CASP9
KCNJ11
ABCB7
ABCG2
ATP7A
KCNE2
SNCA
MIR7-1
MIR24-1
CHRNA7
TLR7
DHFR
DAPK1
FLVCR2
MIRLET7E
PRRT2
TRPM6
GNB3
ADAMTS2
TERT
MIR181B1
SLC6A3
AMACR
MIR214
F2R
MIR100
ITPR1
PIK3CG
MIR27A
MIR374A
ITGB6
BIRC5
GABRG2
PKD1
MAT2A

CYP19A1
FOXE3
MIR93
RBP4
FKTN
MAPK14
AR
HTR2A
XK
PPT1
APOA2
CD34
SCN10A
SAMHD1
FGF5
MIR382
EGR1
MIRLET7D
MIR486-1
MIR15B
PLA2G2A
ADK
CORIN
IGF2
FOLH1
MIR195
MIR22
MIR335
DKK1
ATM
AP3B2
RNF13
CNPY3
TMTC3
EGFR
CHD1L
MYC
OIP5-AS1

LRPPRC
NR4A2
TUBB3
TBC1D1
C3
IDO1
MIRLET7A1
GSR
DPM3
MIR766
SNORD118
CD274
SLC16A1
PDE5A
KL
GAD1
GUSB
MIR204
HDAC4
TAFAZZIN
MIR182
MIR181A1
CCL11
NINJ2
GRM7
CACNA1H
ATG7
TNFRSF9
DMXL2
MYH9
HLA-DRB1
MET
MED12
HNF4A
CASP8
RAI1
TANGO2
SLC6A1

INSR
CYBA
BAX
MIR186
MIR148A
FLNC
PRL
SPG11
OCLN
FOXP2
WNK1
SLC1A1
CTSD
MIR138-1
GHRL
TSC2
ATRX
DCX
MECP2
PXDN
CELSR1
GH1
ASPM
GALC
SGCE
KCNQ1OT1
CASP1
MIR139
MAPK10
PNKD
MIR10A
IL6R
LGALS3
CRYAB
IL4R
TMEM106B
SCN1A
MLYCD

GOSR2
MFAP5
MIR181C
SLC20A2
DARS2
MIR135B
XBP1
DCC
GRM8
MIR296
ITIH4
MIR132
EMSLR
HNRNPH2
CSF1
SP1
STAT5B
MMP12
IGFBP3
FMR1
HMBS
ASAH1
GLS
GCDH
SUOX
MIR34B
IFNA1
GGT1
RHOA
GPX3
F9
CTC1
NTRK1
SLC37A4
PTPN22
ABL1
GCK
TYROBP

ST3GAL3
MIR205
MBP
RNU4ATAC
ETV4
FREM2
CNNM2
GRIN1
MIR20A
CPT2
CLDN5
HSPA5
FUS
GJA4
SPR
HEXA
UCA1
SHANK3
CXCR2
CAT
MIR183
TCF7L2
CD28
DYNC1H1
COG4
MIR30B
SCN2B
MIR151A
FCGR2A
MIR497
ALKBH8
CLCN2
GSK3B
MBL2
CYP2J2
SHH
GSN
MIR377

HSD11B2
PRKRA
MYD88
WT1
PEX1
PIGA
PDP1
B3GLCT
TIMP2
STX1A
NSD1
ATG5
NRG1
MMP8
MIR483
CYP2R1
CSRP3
MIR381
ADA
MB
MIR328
CACNA1B
AARS2
SHOC2
IDUA
BRCA1
CASR
MIR30E
FLT4
ABCA7
RNF216
NGB
NEDD4L
MYL4
MIR23B
ITGB2
SDHD
MIR362

POMC
MIR324
MIR29C
ALDH18A1
IL33
MIR361
NEFL
NCAM1
RFT1
MIR188
MIR25
TFAP2A
RAC1
COL18A1
DYRK1A
GALNT2
ATP6V0A2
KIF5A
ADRA2B
SLC25A15
MMP14
TIMP3
LEPR
MIR103A1
RBM20
SIGMAR1
ST3GAL5
GCKR
CHKB
MFN2
ITLN1
NAGS
ARMS2
FASLG
PVALB
CCL5
KCNA5
GZMB

TRAPPC9
FABP4
MIR197
MIR33A
ELANE
LOC106627981
HNF1A
ATP1A2
MIR200C
OSMR
RYR1
GYS2
KIF11
CYP2C8
KCNB1
NTN1
PLIN1
GABRA2
ERCC6
KMT2A
NDST1
BMP2
FAT4
SPRED1
BMP4
HEY2
GSTP1
JAK1
IDH1
SEC24D
SUCLG1
MIR23A
MEN1
KCND3
SRPX2
FCGR3A
TAT
NLGN1

ANXA2
VIM
SELL
TLR3
MOCS2
KCNH2
IL5RA
MAP2K2
ALDH5A1
SCN9A
MIR455
UGT1A1
TBX18
PROCR
EXTL3
NDUFS4
STAT5A
LARS2
SCN2A
BCL2L11
FUCA1
AGA
NANS
NBAS
TRAF6
BGLAP
ASPA
CD4
TGIF1
IL12A
CUL3
PTX3
MIR330
SCARB2
CPT1A
DSG2
COQ8A
CYP27B1

SLC39A8
SLC25A3
CAMTA1
METTL3
MIR212
SETBP1
MIR491
GLUL
IKBKG
KLF4
TAF1
VCL
NEU1
MC2R
PSPH
COG7
CKB
GNRH1
CDH15
PACS2
ARVCF
SOX17
OPA1
MCCC2
SCN8A
PYGM
PKP2
ASXL2
KIT
G6PC1
CYP3A5
GNPTAB
IRS1
CDR1-AS
GRM1
C5
PIGQ
PCNA

MIR423
MIR92A1
COL5A2
SURF1
ATP6V1A
MIR196A1
GAS5-AS1
GATM
EXT2
MVK
SNHG7
MIR454
GPT2
PEX10
NFIA
GJC2
KCNJ6
ABCG8
SRSF3
MYL3
HADH
MIR101-1
MAFB
TTC21B
WDR19
DNMT1
JMJD1C
RELA
HAVCR2
CCK
CASP7
LYZ
DOCK8
PDYN
MFSD8
EPRS1
SOX9
SLC12A1

MIRLET7C
ALG13
PINK1
WRN
GPC3
POMT2
FOXO1
PROM1
IFNB1
FUT8
ALG12
MYT1L
POLG2
SMARCB1
MIR545
RPIA
HTR3A
ATP5F1A
CFLAR
DOLK
IGF2BP2
MAN2B1
CXCL1
NAGLU
ADORA1
AP3D1
SLC25A4
MIR490
MAP2
CTBP1
COL13A1
SPTBN4
ACOT7
TBCD
PIGG
WDR37
MYO7B
MDH2

MIR106A
CEP164
ERCC2
SLC4A1
CYP2D6
HMGCL
TBX5
GNA11
XIAP
GABRA1
MTPAP
AIF1
LRP2
PRDM8
MIR502
CXCL10
MAP1B
FAM111A
MMP13
TBXA2R
KLKB1
DOCK7
MIR19B1
IL9
NT5E
STAMBP
CCBE1
SNORD15A
PPP3CA
CYP4A11
DIP2B
HDAC3
MIR127
GBE1
KAT2B
FASTKD2
CD86
GP5

CARS2
TFPI
REEP1
FH
ALMS1
PCDH19
TYMS
LYRM7
MIR424
FOXP3
TFRC
ARCN1
FKRP
APAF1
CNR1
CDKN3
FABP3
ATP2A2
CX3CR1
ATP5F1E
TNXB
TBL1XR1
GABRD
SLC9A6
PEX2
MIR340
ANGPT2
DGUOK
PI4KA
ABO
MIR193B
SRCAP
COL11A1
CD8A
NCF1
MIR137
MIR1246
MIR200A

LZTR1
PTCH1
CALCA
COL6A2
TUBGCP6
IL1F10
COL4A5
SCGB1A1
CALM3
MIR378A
MTRFR
CCND1
CCR1
MTHFD1
NAMPT
ANK3
TYR
CHRNB2
IL12B
PGK1
F13B
AQP1
ABAT
MPDZ
MIR375
HSP90AA1
FGF10
MIR136
DNM1L
RMST
SERPIND1
GNAI1
GRIA3
TBX1
ROCK1
TBCE
FGD1
PLK4

LOC106099062
LOC107133510
GTPBP3
MIR31
SCN3A
SORT1
CHD7
HSD17B10
MIR128-1
MIR28
MIR338
GPX4
NLRP12
ADRB3
MYOCD
SPAST
MC4R
FLI1
CLU
CREBBP
MIR590
CHD8
MIR224
TWNK
CCL3
NOL3
UPF3B
NUS1
NR2F1
GABBR1
PIK3CB
TXN2
BCR
TPM1
MIR370
MIR154
SNAP25
ATAD3A

COQ8B
PPP2CA
FGF7
ZEB1
RNLS
ERCC8
SNAP29
MIR506
FBN2
MTM1
PLAU
PCDH7
MIR744
COL9A2
AMPD1
DDC
KATNB1
EDNRB
CRHR1
DPYD
NES
MIR133A1
MYL2
SLC12A5
GCG
AFF2
RRM2B
NDUFS6
EPG5
TMEM126B
DENND2B
CHD3
SOX5
RILP
SMPD1
EBP
PPOX
STAT1

GNB1
VLDLR
ROGDI
PTK2
GSTM1
CACNB4
GABRB1
ANKH
EPHB4
SARS2
MIR17HG
MIR615
ATP6AP2
DNM1
SNX10
MED23
B4GALT7
AXL
EDN3
PAH
CEACAM5
PEX5
PEX16
GLP1R
MIR582
MUC1
MIR302A
PC
COL11A2
MIR30C1
MIR135A1
MIR301A
MIR187
MIR520A
MIR654
MIR876
MIR518A1
MIR363

MIR509-1
MIR625
PIGL
MIR873
MIR513A1
PROS1
IDH2
FGF4
CDKN1A
IL2RA
CRPPA
MIR616
PNPO
RORA
ARNT2
AUTS2
ARNT
KCNA2
GP1BB
TUBB
MCCC1
SNRPN
CEP152
SLC12A2
MSX2
SOX10
CDH11
PXDNL
PHGDH
GNS
CEP290
POGZ
MCEE
QARS1
LAMA2
TRA-TGC7-1
CUX1
RAD21

MMAA
PDCD1
NFU1
SDCCAG8
BOLA3
IBA57
ADD3
NGLY1
KIF1A
RTTN
PIGW
HLCS
TIMM8A
FHL1
CCR3
FMN2
AFP
MIR524
SPTAN1
CHRNA4
KLLN
PCK1
SLC12A3
IL23R
NPR3
EARS2
GCM2
CYP1B1
HAMP
ALDH7A1
EMC1
PPP2R1A
NFKBIA
SUFU
CHIT1
LDHA
CAV3
EZR

IRX2-DT
EED
KIRREL3
STAG1
CTR9
UBE4A
ACER3
PIGU
CHAMP1
DLD
TSC1
BCL11A
GLUD1
PEX6
CACNB2
MIR192
CD47
ALG6
CLPB
NID1
TAC3
JPH2
PDCD4
EPCAM
CARD9
PEX19
KCNE1
PRDX1
FGF9
ERBB2
MIR1207
IRAK1
KCNQ2
KCNA1
ITGAL
PGM1
GNE
MBOAT7

FGF6
TAB2
MMP10
PIGT
PIGV
DNAJC5
HPRT1
SLC18A2
TYMP
PIWIL1
ACADM
PKD2
CDK1
CCL20
TAFA4
ASL
MIR526B
MIR372
MIR383
MYO5A
KIAA0586
PIEZO2
EMD
IDS
LINC-ROR
B3GALT6
MOCS1
HLA-C
DICER1
GNAO1
TCAP
GMPPB
DHDDS
SLC35A2
GABRB3
RAB11A
PAFAH1B1
VAMP2

MYH1
SST
DANCR
CDH2
RBFOX1
GLDC
ETS1
MPV17
CD44
MIR339
TMEM67
SPRY4-IT1
LARS1
ASXL1
DLL4
PQBP1
HIP1
NIN
CKAP2L
MRPL44
MLH1
CYP1A1
ATPAF2
SHBG
SCN11A
WNT5A
CPOX
SDHAF2
ARF1
PGF
HLA-A
LBR
QDPR
POMGNT1
POMT1
TUBA1A
GSS
EFEMP2

EPM2A
CLN5
NHLRC1
GOT2
ACOX1
GK
CLCNKB
CKM
ALG1
IL15
INO80
SCN7A
MIR107
SLCO1B1
H2AX
AGXT
ASNS
HAX1
MSH2
LONP1
POLR3B
AVPR2
STXBP1
ACVR1
COL9A3
PLXND1
BAG3
NDUFAF6
DNAH5
ATP6V0C
CD55
SCO2
MTO1
PUS1
COX10
COX15
COX20
MTFMT

MIR511
MSH6
TNFRSF1B
LARGE1
COG5
PTF1A
IER3IP1
DSCAM
DKC1
ALDOB
GALT
SLC25A13
THAP11
MYPN
CSNK2A1
PEX14
PEX7
PEX3
PEX11B
PEX13
PEX26
PEX12
ASCL1
SLC6A9
MEF2C
MASP2
GAMT
CLPP
PIK3CD
VRK2
ZNF335
FGF3
FGF18
FGF16
FGF20
FGF22
AQP2
TUBB2B

CENPJ
KEAP1
LOC110806262
MIR708
TCF4
NLGN3
ADRA2A
POU1F1
CENPE
RBFOX3
UCP3
BCKDK
SMARCA2
GFER
IGFBP1
AASS
ALDH4A1
SACS
GRIA4
PAX6
MFSD2A
BCS1L
MKS1
PLP1
ZMPSTE24
LIF
HERC2
CAMK2A
ADNP
STK11
TRPS1
SLC5A2
BSCL2
MIR92B
FOXO3
FARS2
NCAPD3
LGALS9

GP9
HNRNPA2B1
PLN
ATIC
ACADS
MARS2
MPC1
NDUFA4
XPNPEP3
SC5D
B4GALT1
HCFC1
MAN1B1
DDOST
ATP6AP1
MGAT2
MPI
DPM1
GFM1
MOGS
ALG8
MPDU1
ALG2
ALG3
ALG9
DNAJC19
MAGT1
MMADHC
SRD5A3
SSR4
TUSC3
GMPPA
NDUFB11
PIGN
PIGO
SLC35A3
SLC35C1
ALG11

B3GALNT2
LMBRD1
PGAP3
PIGM
SLC35A1
TMEM165
COG1
COG6
COG8
MAGEL2
POMGNT2
B4GAT1
CCDC115
DPM2
POMK
RXYLT1
PGAP2
NPHP1
PHOX2B
AGO2
ALX4
GRIA1
SHANK2
DROSHA
SULT1A3
CSTB
MAF
ATN1
CERS1
RPGRIP1L
POLR3A
PHF6
KLRC4
UBAC2
PLK1
GDF15
TRIT1
FGF13

RB1
CIT
KCNJ1
CACNA1E
KCNQ3
PNKP
FOXG1
COPB2
ANKLE2
CDK5RAP2
MCPH1
STIL
WDR62
LTF
APOA4
SMARCE1
RARS2
SAA1
MIR651
PAPPA
APTX
CALM2
WEE1
PIGF
NRP1
ALOX15
SLC22A5
AGK
FOXM1
PLCB1
DLAT
DHCR24
HCCS
PLAGL1
BPNT2
ARFGEF2
CLDN16
ZFP57

C4B
CHEK2
KRT7
CASQ2
COL9A1
STAT2
NODAL
TMEM216
CLTC
SYT14
FLII
PAK1
KCND2
GLI3
SATB2
PROP1
ATP1A1
MS4A2
BMP6
BGN
SCNN1G
FDFT1
RAB3GAP2
ADGRV1
SHROOM4
TGFBR3
GLB1
SLC6A8
TPK1
MIR505
PIK3R2
E2F1
HSD17B4
RBPJ
SUMF1
CNTN4
SETD5
APLNR

DHCR7
ALAD
SCO1
ALDH3A2
KCNJ10
PTS
FA2H
PMPCA
SPG7
LIMK1
AARS1
SYNGAP1
CHD2
ARHGAP31
DNM3
IL1RL1
SERPINF2
POLR1C
WWOX
DNMT3B

Table S2. GO enrichment of the 35 selected targets.

GO Category	Term	Name	Count	Genes	PValue	FDR
Biological Process (139)	GO:0007165	signal transduction	12	CXCL12, CDK6, CSNK2A1, CHRNA7, PDE4D, NLRP3, PDE5A, MAPK14, ESR1, EGFR, ESR2, IGF1R	8.14E-06	7.31E-03
	GO:0050727	regulation of inflammatory response	5	ALOX5, ALOX15, NLRP3, PTGS2, ESR1	2.80E-05	1.18E-02
	GO:0071392	cellular response to estradiol stimulus	4	ESR1, EGFR, ESR2, IGF1R	3.93E-05	1.18E-02
	GO:1900015	regulation of cytokine production involved in inflammatory response	3	NOS2, ALOX5, MAPK14	1.61E-04	2.89E-02
	GO:0010629	negative regulation of gene expression	6	APP, TERT, NOS2, CDK1, ESR1, XDH	1.88E-04	2.89E-02
	GO:0019372	lipoxygenase pathway	3	ALOX5, ALOX15, PTGS2	1.93E-04	2.89E-02
	GO:0043065	positive regulation of apoptotic process	6	APP, IGFBP3, KCNMA1, CYP1B1, PTGS2, SNCA	2.45E-04	3.14E-02
	GO:0071222	cellular response to lipopolysaccharide	5	NOS2, GSTP1, NLRP3, MAPK14, PTGS2	3.37E-04	3.78E-02

GO:0043410	positive regulation of MAPK cascade	5	APP, CHRNA7, IGFBP3, EGFR, IGF1R	4.24E-04	4.04E-02
GO:0032355	response to estradiol	4	CYP1B1, PTGS2, ESR1, CYP19A1	4.50E-04	4.04E-02
GO:0042759	long-chain fatty acid biosynthetic process	3	ALOX5, ALOX15, PTGS2	6.08E-04	4.55E-02
GO:0032930	positive regulation of superoxide anion generation	3	APP, GSTP1, EGFR	6.08E-04	4.55E-02
GO:2000379	positive regulation of reactive oxygen species metabolic process	3	CYP1B1, MAPK14, XDH	1.70E-03	1.17E-01
GO:0019369	arachidonate metabolic process	3	ALOX5, ALOX15, CYP1B1	2.10E-03	1.35E-01
GO:0042307	positive regulation of protein import into nucleus	3	CDK1, MAPK14, PTGS2	2.55E-03	1.39E-01
GO:0045766	positive regulation of angiogenesis	4	TERT, CHRNA7, CYP1B1, F3	2.75E-03	1.39E-01
GO:0032691	negative regulation of interleukin-1 beta production	3	CHRNA7, GSTP1, NLRP3	2.79E-03	1.39E-01
GO:0033138	positive regulation of peptidyl-serine phosphorylation	3	APP, EGFR, SNCA	2.79E-03	1.39E-01

GO:0045429	positive regulation of nitric oxide biosynthetic process	3	APP, PTGS2, ESR1	3.04E-03	1.44E-01
GO:0001666	response to hypoxia	4	CXCL12, NOS2, CHRNA7, KCNMA1	3.65E-03	1.45E-01
GO:0008344	adult locomotory behavior	3	APP, CXCL12, SNCA	3.71E-03	1.45E-01
GO:0000086	G2/M transition of mitotic cell cycle	3	APP, ABCB1, CDK1	3.71E-03	1.45E-01
GO:0048146	positive regulation of fibroblast proliferation	3	CDK6, ESR1, EGFR	3.71E-03	1.45E-01
GO:0048661	positive regulation of smooth muscle cell proliferation	3	PTGS2, EGFR, IGF1R	4.00E-03	1.50E-01
GO:0006915	apoptotic process	6	APP, CSNK2A1, IGFBP3, CDK1, NLRP3, MAPK14	5.29E-03	1.85E-01
GO:0050808	synapse organization	3	APP, CHRNA7, SNCA	5.40E-03	1.85E-01
GO:0070374	positive regulation of ERK1 and ERK2 cascade	4	APP, CHRNA7, ALOX15, EGFR	5.55E-03	1.85E-01
GO:0007611	learning or memory	3	APP, CHRNA7, EGFR	6.09E-03	1.95E-01

GO:0008285	negative regulation of cell population proliferation	5	APP, CDK6, IGFBP3, CYP1B1, PTGS2	6.59E-03	2.04E-01
GO:1905906	regulation of amyloid fibril formation	2	APP, CHRNA7	6.96E-03	2.08E-01
GO:0018105	peptidyl-serine phosphorylation	3	CSNK2A1, CDK1, MAPK14	7.97E-03	2.30E-01
GO:0043124	negative regulation of canonical NF-kappaB signal transduction	3	CHRNA7, GSTP1, ESR1	8.38E-03	2.30E-01
GO:0071260	cellular response to mechanical stimulus	3	PTGS2, EGFR, IGF1R	8.58E-03	2.30E-01
GO:0032310	prostaglandin secretion	2	NOS2, PTGS2	8.70E-03	2.30E-01
GO:0009410	response to xenobiotic stimulus	4	ABCB1, CDK1, PTGS2, SNCA	9.84E-03	2.40E-01
GO:0010628	positive regulation of gene expression	5	APP, CDK6, CDK1, MAPK14, F3	1.00E-02	2.40E-01
GO:0007613	memory	3	APP, CHRNA7, PTGS2	1.01E-02	2.40E-01
GO:1990962	xenobiotic transport across blood-brain barrier	2	ABCB1, ABCG2	1.04E-02	2.40E-01

GO:0006527	arginine catabolic process	2	NOS2, ARG1	1.04E-02	2.40E-01
GO:0043066	negative regulation of apoptotic process	5	GSTP1, CDK1, EGFR, IGF1R, SNCA	1.20E-02	2.43E-01
GO:0071393	cellular response to progesterone stimulus	2	CYP1B1, IGF1R	1.22E-02	2.43E-01
GO:0034440	lipid oxidation	2	ALOX5, ALOX15	1.22E-02	2.43E-01
GO:0002674	negative regulation of acute inflammatory response	2	GSTP1, NLRP3	1.22E-02	2.43E-01
GO:0098815	modulation of excitatory postsynaptic potential	2	APP, CHRNA7	1.22E-02	2.43E-01
GO:0098586	cellular response to virus	3	MMP12, NLRP3, MAPK14	1.22E-02	2.43E-01
GO:0030335	positive regulation of cell migration	4	CXCL12, F3, EGFR, IGF1R	1.36E-02	2.65E-01
GO:0051122	hepoxilin biosynthetic process	2	GSTP1, ALOX15	1.39E-02	2.65E-01
GO:1903426	regulation of reactive oxygen species biosynthetic process	2	ALOX5, SNCA	1.56E-02	2.92E-01

GO:0050729	positive regulation of inflammatory response	3	APP, NLRP3, SNCA	1.63E-02	2.94E-01
GO:0045944	positive regulation of transcription by RNA polymerase II	7	MMP12, APP, NLRP3, MAPK14, ESR1, EGFR, ESR2	1.68E-02	2.94E-01
GO:0009624	response to nematode	2	ARG1, PTGS2	1.73E-02	2.94E-01
GO:0070633	transepithelial transport	2	ABCB1, ABCG2	1.73E-02	2.94E-01
GO:0140115	export across plasma membrane	2	ABCB1, ABCG2	1.73E-02	2.94E-01
GO:0006805	xenobiotic metabolic process	3	ABCB1, GSTP1, CYP1B1	1.92E-02	3.19E-01
GO:0001934	positive regulation of protein phosphorylation	3	APP, CHRNA7, EGFR	2.03E-02	3.19E-01
GO:0034121	regulation of toll-like receptor signaling pathway	2	APP, ESR1	2.08E-02	3.19E-01
GO:0050730	regulation of peptidyl-tyrosine phosphorylation	2	APP, EGFR	2.08E-02	3.19E-01
GO:0032754	positive regulation of interleukin-5 production	2	PDE4D, NLRP3	2.08E-02	3.19E-01

GO:0006979	response to oxidative stress	3	APP, PTGS2, EGFR	2.10E-02	3.19E-01
GO:0071872	cellular response to epinephrine stimulus	2	PDE4D, SNCA	2.25E-02	3.31E-01
GO:0038083	peptidyl-tyrosine autophosphorylation	2	EGFR, IGF1R	2.25E-02	3.31E-01
GO:0051000	positive regulation of nitric-oxide synthase activity	2	TERT, ESR1	2.42E-02	3.45E-01
GO:0000122	negative regulation of transcription by RNA polymerase II	6	MMP12, APP, CDK6, ESR1, ESR2, SNCA	2.42E-02	3.45E-01
GO:0044849	estrous cycle	2	CYP1B1, IGF1R	2.76E-02	3.64E-01
GO:1904645	response to amyloid-beta	2	MMP12, CHRNA7	2.76E-02	3.64E-01
GO:0048169	regulation of long-term neuronal synaptic plasticity	2	APP, SNCA	2.76E-02	3.64E-01
GO:0019395	fatty acid oxidation	2	ALOX15, MAPK14	2.76E-02	3.64E-01
GO:0090336	positive regulation of brown fat cell differentiation	2	MAPK14, PTGS2	2.76E-02	3.64E-01

GO:0050728	negative regulation of inflammatory response	3	ALOX5, CHRNA7, NLRP3	2.95E-02	3.84E-01
GO:0016125	sterol metabolic process	2	CYP1B1, CYP19A1	3.10E-02	3.92E-01
GO:0048143	astrocyte activation	2	APP, EGFR	3.10E-02	3.92E-01
GO:0071391	cellular response to estrogen stimulus	2	ESR1, ESR2	3.27E-02	3.96E-01
GO:0060065	uterus development	2	ESR1, CYP19A1	3.27E-02	3.96E-01
GO:0033280	response to vitamin D	2	TYR, PTGS2	3.27E-02	3.96E-01
GO:0043651	linoleic acid metabolic process	2	GSTP1, ALOX15	3.44E-02	4.11E-01
GO:2000406	positive regulation of T cell migration	2	APP, CXCL12	3.77E-02	4.46E-01
GO:0030520	estrogen receptor signaling pathway	2	ESR1, ESR2	3.94E-02	4.48E-01
GO:1900273	positive regulation of long-term synaptic potentiation	2	APP, CHRNA7	3.94E-02	4.48E-01
GO:0090026	positive regulation of monocyte chemotaxis	2	APP, CXCL12	3.94E-02	4.48E-01

GO:0006809	nitric oxide biosynthetic process	2	NOS2, CYP1B1	4.11E-02	4.61E-01
GO:0046686	response to cadmium ion	2	TERT, CDK1	4.28E-02	4.74E-01
GO:2000773	negative regulation of cellular senescence	2	CDK6, TERT	4.44E-02	4.81E-01
GO:0051247	positive regulation of protein metabolic process	2	APP, CHRNA7	4.44E-02	4.81E-01
GO:0051262	protein tetramerization	2	APP, SNCA	4.78E-02	4.93E-01
GO:0010971	positive regulation of G2/M transition of mitotic cell cycle	2	APP, CDK1	4.78E-02	4.93E-01
GO:0051897	positive regulation of phosphatidylinositol 3-kinase/protein kinase B signal transduction	3	APP, EGFR, IGF1R	4.92E-02	4.93E-01
GO:0001774	microglial cell activation	2	APP, SNCA	4.94E-02	4.93E-01
GO:0095500	acetylcholine receptor signaling pathway	2	ACHE, CHRNA7	4.94E-02	4.93E-01
GO:0019933	cAMP-mediated signaling	2	PDE4D, PDE5A	4.94E-02	4.93E-01

GO:1905606	regulation of presynapse assembly	2	APP, SNCA	4.94E-02	4.93E-01
GO:0071549	cellular response to dexamethasone stimulus	2	EGFR, IGF1R	5.11E-02	4.99E-01
GO:1902894	negative regulation of miRNA transcription	2	APP, ESR1	5.11E-02	4.99E-01
GO:0045907	positive regulation of vasoconstriction	2	PTGS2, EGFR	5.28E-02	5.09E-01
GO:1990000	amyloid fibril formation	2	APP, SNCA	5.44E-02	5.09E-01
GO:0045740	positive regulation of DNA replication	2	CDK1, EGFR	5.44E-02	5.09E-01
GO:0010575	positive regulation of vascular endothelial growth factor production	2	CYP1B1, PTGS2	5.44E-02	5.09E-01
GO:0051968	positive regulation of synaptic transmission, glutamatergic	2	PTGS2, EGFR	5.61E-02	5.14E-01
GO:0060045	positive regulation of cardiac muscle cell proliferation	2	CDK1, MAPK14	5.61E-02	5.14E-01
GO:2000463	positive regulation of excitatory postsynaptic potential	2	APP, CHRNA7	5.77E-02	5.24E-01

GO:0008284	positive regulation of cell population proliferation	4	CSNK2A1, CHRNA7, EGFR, IGF1R	5.92E-02	5.28E-01
GO:0008306	associative learning	2	APP, CHRNA7	5.94E-02	5.28E-01
GO:0070555	response to interleukin-1	2	APP, SNCA	6.10E-02	5.37E-01
GO:0031100	animal organ regeneration	2	CXCL12, CDK1	6.27E-02	5.41E-01
GO:0046326	positive regulation of D-glucose import	2	TERT, MAPK14	6.27E-02	5.41E-01
GO:0001937	negative regulation of endothelial cell proliferation	2	ALOX5, XDH	6.59E-02	5.64E-01
GO:0018107	peptidyl-threonine phosphorylation	2	CSNK2A1, CDK1	6.76E-02	5.72E-01
GO:0048144	fibroblast proliferation	2	CDK1, ESR1	6.92E-02	5.81E-01
GO:0035094	response to nicotine	2	CHRNA7, IGF1R	7.08E-02	5.89E-01
GO:0007155	cell adhesion	4	ACHE, APP, CXCL12, CYP1B1	7.30E-02	6.02E-01
GO:0030177	positive regulation of Wnt signaling pathway	2	CSNK2A1, TERT	7.41E-02	6.05E-01

GO:0006469	negative regulation of protein kinase activity	2	GSTP1, SNCA	7.57E-02	6.09E-01
GO:0050796	regulation of insulin secretion	2	NOS2, ALOX5	7.73E-02	6.09E-01
GO:0042177	negative regulation of protein catabolic process	2	NOS2, EGFR	7.73E-02	6.09E-01
GO:0045663	positive regulation of myoblast differentiation	2	IGFBP3, MAPK14	7.73E-02	6.09E-01
GO:0001525	angiogenesis	3	CYP1B1, MAPK14, PTGS2	7.89E-02	6.11E-01
GO:1904646	cellular response to amyloid-beta	2	APP, IGF1R	7.89E-02	6.11E-01
GO:0001933	negative regulation of protein phosphorylation	2	IGFBP3, XDH	8.05E-02	6.18E-01
GO:0036323	vascular endothelial growth factor receptor-1 signaling pathway	2	EGFR, IGF1R	8.21E-02	6.18E-01
GO:0042130	negative regulation of T cell proliferation	2	ARG1, PDE5A	8.37E-02	6.18E-01
GO:0043409	negative regulation of MAPK cascade	2	GSTP1, IGF1R	8.37E-02	6.18E-01

GO:0035790	platelet-derived growth factor receptor-alpha signaling pathway	2	EGFR, IGF1R	8.37E-02	6.18E-01
GO:0038109	Kit signaling pathway	2	EGFR, IGF1R	8.54E-02	6.18E-01
GO:0038145	macrophage colony-stimulating factor signaling pathway	2	EGFR, IGF1R	8.54E-02	6.18E-01
GO:0042311	vasodilation	2	KCNMA1, EGFR	8.54E-02	6.18E-01
GO:0006468	protein phosphorylation	3	CDK6, CSNK2A1, IGFBP3	8.66E-02	6.22E-01
GO:1900087	positive regulation of G1/S transition of mitotic cell cycle	2	TERT, EGFR	8.86E-02	6.23E-01
GO:0031547	brain-derived neurotrophic factor receptor signaling pathway	2	EGFR, IGF1R	9.01E-02	6.23E-01
GO:0051592	response to calcium ion	2	KCNMA1, EGFR	9.01E-02	6.23E-01
GO:0048863	stem cell differentiation	2	MAPK14, ESR1	9.01E-02	6.23E-01
GO:0035791	platelet-derived growth factor receptor-beta signaling pathway	2	EGFR, IGF1R	9.01E-02	6.23E-01

GO:0048012	hepatocyte growth factor receptor signaling pathway	2	EGFR, IGF1R	9.33E-02	6.35E-01
GO:0031623	receptor internalization	2	ACHE, SNCA	9.33E-02	6.35E-01
GO:0071320	cellular response to cAMP	2	PDE4D, CYP1B1	9.49E-02	6.36E-01
GO:0045786	negative regulation of cell cycle	2	CDK6, PTGS2	9.49E-02	6.36E-01
GO:0050890	cognition	2	APP, CHRNA7	9.65E-02	6.37E-01
GO:0038063	collagen-activated tyrosine kinase receptor signaling pathway	2	EGFR, IGF1R	9.65E-02	6.37E-01
GO:0030282	bone mineralization	2	ALOX15, PTGS2	9.81E-02	6.38E-01
GO:0090398	cellular senescence	2	MAPK14, IGF1R	9.81E-02	6.38E-01
GO:0007612	learning	2	APP, PTGS2	9.97E-02	6.44E-01

Category	Term	Name	Count	Genes	PValue	FDR
Cell Component (27)	GO:0048471	perinuclear region of cytoplasm	8	ACHE, APP, NOS2, ALOX5, PDE4D, TYR, EGFR, SNCA	1.77E-04	2.30E-02
	GO:0005829	cytosol	20	APP, NOS2, CSNK2A1, MAOA, ARG1, GSTP1, PDE4D, ALOX15, PTGS2, MAPK14, ESR1, EGFR, CDK6, TERT, ALOX5, CDK1, NLRP3, PDE5A, XDH, SNCA	4.46E-04	2.30E-02
	GO:0016324	apical plasma membrane	6	ABCB1, CHRNA7, PDE4D, KCNMA1, EGFR, ABCG2	5.28E-04	2.30E-02
	GO:0005615	extracellular space	11	MMP12, ACHE, APP, ARG1, ALOX5, GSTP1, IGFBP3, F3, XDH, EGFR, SNCA	5.82E-04	2.30E-02
	GO:0005739	mitochondrion	10	APP, ALDH2, MAOA, GSTP1, CDK1, NLRP3, CYP1B1, MAPK14, ESR2, SNCA	1.39E-03	4.41E-02
	GO:0005576	extracellular region	11	MMP12, ACHE, APP, CXCL12, ARG1, ALOX5, GSTP1, IGFBP3, NLRP3, MAPK14, SNCA	3.01E-03	7.94E-02
	GO:0005901	caveola	3	KCNMA1, PTGS2, IGF1R	6.68E-03	1.51E-01
	GO:0098591	external side of apical plasma membrane	2	ABCB1, ABCG2	9.77E-03	1.93E-01

GO:0005634	nucleus	18	ACHE, APP, NOS2, CSNK2A1, ARG1, GSTP1, PDE4D, IGFBP3, MAPK14, ESR1, EGFR, ESR2, MMP12, CDK6, TERT, CDK1, NLRP3, SNCA	1.14E-02	2.00E-01
GO:0034774	secretory granule lumen	3	ALOX5, GSTP1, MAPK14	1.59E-02	2.19E-01
GO:0005641	nuclear envelope lumen	2	APP, ALOX5	1.62E-02	2.19E-01
GO:0016020	membrane	16	ACHE, APP, ABCB1, CHRNA7, PDE4D, ALOX15, F3, ESR1, CYP19A1, EGFR, IGF1R, KCNMA1, CDK1, CYP1B1, NLRP3, SNCA	1.69E-02	2.19E-01
GO:1904813	ficolin-1-rich granule lumen	3	ALOX5, GSTP1, MAPK14	1.80E-02	2.19E-01
GO:0009986	cell surface	5	ACHE, APP, ABCB1, F3, EGFR	2.21E-02	2.27E-01
GO:0005886	plasma membrane	16	ACHE, APP, ABCB1, NOS2, CSNK2A1, CHRNA7, PDE4D, ALOX15, F3, ESR1, EGFR, IGF1R, TERT, KCNMA1, ABCG2, SNCA	2.28E-02	2.27E-01
GO:0043231	intracellular membrane-bounded organelle	6	APP, CYP1B1, TYR, PTGS2, ESR2, IGF1R	2.42E-02	2.27E-01
GO:0032991	protein-containing complex	5	APP, PTGS2, ESR1, EGFR, SNCA	2.54E-02	2.27E-01

GO:0005798	Golgi-associated vesicle	2	APP, TYR	2.59E-02	2.27E-01
GO:0098794	postsynapse	3	APP, CHRNA7, SNCA	4.15E-02	3.29E-01
GO:0005737	cytoplasm	16	APP, ABCB1, NOS2, ARG1, GSTP1, TYR, PTGS2, MAPK14, ESR1, EGFR, MMP12, CDK6, TERT, CDK1, NLRP3, SNCA	4.17E-02	3.29E-01
GO:0045121	membrane raft	3	APP, EGFR, ABCG2	4.65E-02	3.50E-01
GO:0043235	receptor complex	3	APP, EGFR, IGF1R	4.89E-02	3.51E-01
GO:0045202	synapse	4	ACHE, APP, CHRNA7, SNCA	5.47E-02	3.76E-01
GO:0000307	cyclin-dependent protein kinase holoenzyme complex	2	CDK6, CDK1	7.26E-02	4.66E-01
GO:0031965	nuclear membrane	3	ALOX5, PDE4D, EGFR	7.37E-02	4.66E-01
GO:0005764	lysosome	3	APP, TYR, SNCA	9.50E-02	5.56E-01
GO:0005788	endoplasmic reticulum lumen	3	APP, IGFBP3, PTGS2	9.50E-02	5.56E-01

Category	Term	Name	Count	Genes	PValue	FDR
Molecular Function (63)	GO:0042803	protein homodimerization activity	9	ACHE, APP, TERT, NOS2, CHRNA7, TYR, PTGS2, XDH, ABCG2	3.88E-05	6.65E-03
	GO:0019899	enzyme binding	7	APP, PDE4D, MAPK14, PTGS2, ESR1, EGFR, ESR2	4.45E-05	6.65E-03
	GO:0005506	iron ion binding	5	ALOX5, ALOX15, CYP1B1, CYP19A1, XDH	1.20E-04	1.20E-02
	GO:0016491	oxidoreductase activity	5	MAOA, ALOX5, TYR, XDH, SNCA	6.94E-04	4.33E-02
	GO:0042802	identical protein binding	11	APP, CSNK2A1, TERT, KCNMA1, NLRP3, TYR, ESR1, EGFR, ABCG2, IGF1R, SNCA	7.24E-04	4.33E-02
	GO:0020037	heme binding	4	NOS2, CYP1B1, PTGS2, CYP19A1	2.60E-03	1.29E-01
	GO:0016701	oxidoreductase activity, acting on single donors with incorporation of molecular oxygen	2	ALOX5, PTGS2	3.53E-03	1.51E-01
	GO:0005524	ATP binding	9	ABCB1, CDK6, CSNK2A1, CDK1, NLRP3, MAPK14, EGFR, ABCG2, IGF1R	4.66E-03	1.55E-01

GO:0005515	protein binding	32	APP, ACHE, ABCB1, MAOA, GSTP1, CHRNA7, ALOX15, PTGS2, EGFR, IGF1R, TERT, ALOX5, CYP1B1, NLRP3, XDH, SNCA, NOS2, CSNK2A1, ARG1, PDE4D, IGFBP3, TYR, MAPK14, F3, ESR1, ESR2, CXCL12, CDK6, KCNMA1, CDK1, PDE5A, ABCG2	4.67E-03	1.55E-01
GO:0050660	flavin adenine dinucleotide binding	3	NOS2, MAOA, XDH	5.87E-03	1.76E-01
GO:0030284	nuclear estrogen receptor activity	2	ESR1, ESR2	7.05E-03	1.76E-01
GO:0004052	arachidonate 12(S)-lipoxygenase activity	2	ALOX5, ALOX15	7.05E-03	1.76E-01
GO:0051117	ATPase binding	3	PDE4D, ESR1, EGFR	1.01E-02	2.32E-01
GO:0101021	estrogen 2-hydroxylase activity	2	CYP1B1, CYP19A1	1.58E-02	3.26E-01
GO:0034056	estrogen response element binding	2	ESR1, ESR2	1.93E-02	3.26E-01
GO:0042166	acetylcholine binding	2	ACHE, CHRNA7	1.93E-02	3.26E-01
GO:0016702	oxidoreductase activity, acting on single donors with incorporation of molecular oxygen, incorporation of two atoms of oxygen	2	ALOX5, PTGS2	2.10E-02	3.26E-01

GO:0043565	sequence-specific DNA binding	4	MMP12, NLRP3, ESR1, ESR2	2.20E-02	3.26E-01
GO:0005520	insulin-like growth factor binding	2	IGFBP3, IGF1R	2.27E-02	3.26E-01
GO:0031994	insulin-like growth factor I binding	2	IGFBP3, IGF1R	2.27E-02	3.26E-01
GO:0015562	efflux transmembrane transporter activity	2	ABCB1, ABCG2	2.44E-02	3.26E-01
GO:0008559	ABC-type xenobiotic transporter activity	2	ABCB1, ABCG2	2.44E-02	3.26E-01
GO:0008395	steroid hydroxylase activity	2	CYP1B1, CYP19A1	2.62E-02	3.26E-01
GO:0106310	protein serine kinase activity	4	CDK6, CSNK2A1, CDK1, MAPK14	2.62E-02	3.26E-01
GO:0004114	3',5'-cyclic-nucleotide phosphodiesterase activity	2	PDE4D, PDE5A	2.79E-02	3.34E-01
GO:0005178	integrin binding	3	APP, CXCL12, EGFR	3.24E-02	3.72E-01
GO:0047555	3',5'-cyclic-GMP phosphodiesterase activity	2	PDE4D, PDE5A	3.65E-02	4.04E-01
GO:0004115	3',5'-cyclic-AMP phosphodiesterase activity	2	PDE4D, PDE5A	3.82E-02	4.07E-01

GO:0005158	insulin receptor binding	2	APP, IGF1R	3.99E-02	4.11E-01
GO:0003707	nuclear steroid receptor activity	2	ESR1, ESR2	4.16E-02	4.14E-01
GO:0042910	xenobiotic transmembrane transporter activity	2	ABCB1, ABCG2	4.33E-02	4.17E-01
GO:0004693	cyclin-dependent protein serine/threonine kinase activity	2	CDK6, CDK1	5.00E-02	4.62E-01
GO:0005496	steroid binding	2	ESR1, ESR2	5.34E-02	4.62E-01
GO:0005516	calmodulin binding	3	NOS2, ESR1, EGFR	5.38E-02	4.62E-01
GO:0003682	chromatin binding	4	APP, CDK1, ESR1, EGFR	5.51E-02	4.62E-01
GO:0030332	cyclin binding	2	CDK6, CDK1	5.84E-02	4.62E-01
GO:0046872	metal ion binding	10	TERT, NOS2, ARG1, PDE4D, IGFBP3, KCNMA1, PDE5A, TYR, PTGS2, SNCA	6.02E-02	4.62E-01
GO:0016301	kinase activity	3	CSNK2A1, CDK1, EGFR	6.07E-02	4.62E-01
GO:0016712	oxidoreductase activity, acting on paired donors, with incorporation or reduction of	2	CYP1B1, CYP19A1	6.17E-02	4.62E-01

	molecular oxygen, reduced flavin or flavoprotein as one donor, and incorporation of one atom of oxygen				
GO:0042626	ATPase-coupled transmembrane transporter activity	2	ABCB1, ABCG2	6.50E-02	4.62E-01
GO:0140359	ABC-type transporter activity	2	ABCB1, ABCG2	7.49E-02	4.62E-01
GO:0001223	transcription coactivator binding	2	TERT, ESR1	8.31E-02	4.62E-01
GO:0005011	macrophage colony-stimulating factor receptor activity	2	EGFR, IGF1R	8.63E-02	4.62E-01
GO:0038062	protein tyrosine kinase collagen receptor activity	2	EGFR, IGF1R	8.63E-02	4.62E-01
GO:0005008	hepatocyte growth factor receptor activity	2	EGFR, IGF1R	8.63E-02	4.62E-01
GO:0005004	GPI-linked ephrin receptor activity	2	EGFR, IGF1R	8.63E-02	4.62E-01
GO:0060175	brain-derived neurotrophic factor receptor activity	2	EGFR, IGF1R	8.63E-02	4.62E-01

GO:0005018	platelet-derived growth factor alpha-receptor activity	2	EGFR, IGF1R	8.63E-02	4.62E-01
GO:0008288	boss receptor activity	2	EGFR, IGF1R	8.63E-02	4.62E-01
GO:0036332	placental growth factor receptor activity	2	EGFR, IGF1R	8.63E-02	4.62E-01
GO:0005009	insulin receptor activity	2	EGFR, IGF1R	8.63E-02	4.62E-01
GO:0005020	stem cell factor receptor activity	2	EGFR, IGF1R	8.63E-02	4.62E-01
GO:0005019	platelet-derived growth factor beta-receptor activity	2	EGFR, IGF1R	8.79E-02	4.62E-01
GO:0005006	epidermal growth factor receptor activity	2	EGFR, IGF1R	8.79E-02	4.62E-01
GO:0030544	Hsp70 protein binding	2	CDK1, SNCA	8.79E-02	4.62E-01
GO:0005007	fibroblast growth factor receptor activity	2	EGFR, IGF1R	8.79E-02	4.62E-01
GO:0005010	insulin-like growth factor receptor activity	2	EGFR, IGF1R	8.95E-02	4.62E-01
GO:0004879	nuclear receptor activity	2	ESR1, ESR2	9.12E-02	4.62E-01

GO:0005021	vascular endothelial growth factor receptor activity	2	EGFR, IGF1R	9.12E-02	4.62E-01
GO:0005005	transmembrane-ephrin receptor activity	2	EGFR, IGF1R	9.28E-02	4.62E-01
GO:0016705	oxidoreductase activity, acting on paired donors, with incorporation or reduction of molecular oxygen	2	CYP1B1, CYP19A1	9.92E-02	4.71E-01
GO:0005507	copper ion binding	2	TYR, SNCA	9.92E-02	4.71E-01
GO:0004672	protein kinase activity	3	CDK1, MAPK14, EGFR	9.97E-02	4.71E-01

Table S3. KEGG pathway annotation of the 35 selected targets.

**Term**	**Name**	**Count**	**Genes**	**PValue**	**FDR**
hsa04913	Ovarian steroidogenesis	5	ALOX5, CYP1B1, PTGS2, CYP19A1, IGF1R	4.40E-05	0.008011063
hsa05200	Pathways in cancer	10	CXCL12, CDK6, TERT, NOS2, GSTP1, PTGS2, ESR1, EGFR, ESR2, IGF1R	1.35E-04	0.012254945

hsa01522	Endocrine resistance	5	MAPK14, ESR1, EGFR, ESR2, IGF1R	5.39E-04	0.032675332
hsa00330	Arginine and proline metabolism	4	ALDH2, NOS2, MAOA, ARG1	9.12E-04	0.034473305
hsa04726	Serotonergic synapse	5	APP, MAOA, ALOX5, ALOX15, PTGS2	9.47E-04	0.034473305
hsa05224	Breast cancer	5	CDK6, ESR1, EGFR, ESR2, IGF1R	2.40E-03	0.072909346
hsa04148	Efferocytosis	5	ARG1, ALOX5, ALOX15, MAPK14, PTGS2	2.98E-03	0.077411552
hsa05225	Hepatocellular carcinoma	5	CDK6, TERT, GSTP1, EGFR, IGF1R	3.96E-03	0.09015345
hsa05207	Chemical carcinogenesis - receptor activation	5	CHRNA7, CYP1B1, ESR1, EGFR, ESR2	9.04E-03	0.158705122
hsa05022	Pathways of neurodegeneration - multiple diseases	7	APP, CSNK2A1, NOS2, CHRNA7, MAPK14, PTGS2, SNCA	9.55E-03	0.158705122
hsa05163	Human cytomegalovirus infection	5	CXCL12, CDK6, MAPK14, PTGS2, EGFR	1.07E-02	0.158705122
hsa01100	Metabolic pathways	13	NOS2, MAOA, ARG1, GSTP1, PDE4D, ALOX15, TYR, PTGS2, CYP19A1, ALDH2, ALOX5, PDE5A, XDH	1.12E-02	0.158705122

hsa00380	Tryptophan metabolism	3	ALDH2, MAOA, CYP1B1	1.13E-02	0.158705122
hsa05010	Alzheimer disease	6	APP, CSNK2A1, NOS2, CHRNA7, PTGS2, SNCA	1.62E-02	0.210569611
hsa04218	Cellular senescence	4	CDK6, IGFBP3, CDK1, MAPK14	2.23E-02	0.270092258
hsa00590	Arachidonic acid metabolism	3	ALOX5, ALOX15, PTGS2	2.45E-02	0.278125729
hsa05204	Chemical carcinogenesis - DNA adducts	3	GSTP1, CYP1B1, PTGS2	3.05E-02	0.281544359
hsa04917	Prolactin signaling pathway	3	MAPK14, ESR1, ESR2	3.05E-02	0.281544359
hsa05206	MicroRNAs in cancer	5	ABCB1, CDK6, CYP1B1, PTGS2, EGFR	3.11E-02	0.281544359
hsa05218	Melanoma	3	CDK6, EGFR, IGF1R	3.21E-02	0.281544359
hsa04115	p53 signaling pathway	3	CDK6, IGFBP3, CDK1	3.38E-02	0.281544359
hsa05214	Glioma	3	CDK6, EGFR, IGF1R	3.46E-02	0.281544359
hsa05133	Pertussis	3	NOS2, NLRP3, MAPK14	3.63E-02	0.281544359

hsa05140	Leishmaniasis	3	NOS2, MAPK14, PTGS2	3.71E-02	0.281544359
hsa05205	Proteoglycans in cancer	4	MAPK14, ESR1, EGFR, IGF1R	4.34E-02	0.316093483
hsa05235	PD-L1 expression and PD-1 checkpoint pathway in cancer	3	CSNK2A1, MAPK14, EGFR	4.70E-02	0.324102758
hsa05222	Small cell lung cancer	3	CDK6, NOS2, PTGS2	4.99E-02	0.324102758
hsa04520	Adherens junction	3	CSNK2A1, EGFR, IGF1R	4.99E-02	0.324102758
hsa05215	Prostate cancer	3	GSTP1, EGFR, IGF1R	5.47E-02	0.343569933
hsa04625	C-type lectin receptor signaling pathway	3	NLRP3, MAPK14, PTGS2	6.19E-02	0.363136938
hsa04064	NF-kappa B signaling pathway	3	CXCL12, CSNK2A1, PTGS2	6.19E-02	0.363136938
hsa04066	HIF-1 signaling pathway	3	NOS2, EGFR, IGF1R	6.71E-02	0.370747484
hsa04914	Progesterone-mediated oocyte maturation	3	CDK1, MAPK14, IGF1R	6.82E-02	0.370747484

hsa05145	Toxoplasmosis	3	NOS2, ALOX5, MAPK14	6.93E-02	0.370747484
hsa00340	Histidine metabolism	2	ALDH2, MAOA	8.18E-02	0.425367595
hsa00220	Arginine biosynthesis	2	NOS2, ARG1	8.54E-02	0.428870767
hsa00230	Purine metabolism	3	PDE4D, PDE5A, XDH	8.72E-02	0.428870767
hsa04926	Relaxin signaling pathway	3	NOS2, MAPK14, EGFR	8.95E-02	0.428870767
hsa04068	FoxO signaling pathway	3	MAPK14, EGFR, IGF1R	9.31E-02	0.434383137
